# How Can We Improve Personal Care Interactions to Reduce Care Refusals From People With Dementia? A Realist Synthesis

**DOI:** 10.1111/jan.70204

**Published:** 2025-09-18

**Authors:** Tamara Backhouse, Anne Killett, Reed W. R. Bratches, Eneida Mioshi

**Affiliations:** ^1^ School of Health Sciences University of East Anglia Norwich Norfolk UK; ^2^ School of Nursing University of Alabama Birmingham Alabama USA

**Keywords:** aberrant motor behaviour in dementia, care refusal in dementia, caregiver, dementia, hygiene, nursing, personal care, realist, safety, trust

## Abstract

**Aim:**

To identify strategies and mechanisms of interventions between caregivers and people with dementia that contribute to reducing refusals of care and determine how they work, in which contexts, why and for whom.

**Design:**

Realist synthesis.

**Methods:**

There were three stages: (1) initial programme theory development and prioritisation through assessing video‐recorded personal care interactions and interview transcripts; scoping the literature and team discussions, (2) literature search, review and synthesis and (3) realist interviews with stakeholders and refinement of evidence‐based programme theories.

**Data Sources:**

Searches were conducted in MEDLINE, EMBASE, PsycINFO, CINAHL Ultimate, Cochrane CENTRAL Register of Controlled Trials and Web of Science; date range: 2000–2024.

**Results:**

A total of 71 sources were included in the synthesis, and interviews with 15 stakeholders. Eight programme theories were generated, evidenced and refined, each incorporating multiple caregiver strategies. The overarching mechanism which made people with dementia more likely to accept assistance with personal care was trusting the caregiver and feeling safe. Seven mechanisms fed into this: a sense of control, positive connection, care feeling manageable, working together, engaging with the care activity (or something non‐care related), comfort and needs being known and addressed.

**Conclusion:**

Refusals of care from people with dementia can be reduced by multiple caregiver strategies related to communication, approach, the type of care offered and the care interaction process. Mechanisms reflect relational aspects: the quality of the caregiver/person partnership and making the person with dementia feel safe.

**Practice Implications:**

Our findings provide programme theories and practical care strategies which could be helpful for those, such as nurses, working to improve personal care practices for people with dementia.

**Patient Contributions:**

Public representatives advised the study throughout, providing advice on initial programme theories, evidence‐based programme theories and synthesised stakeholder evidence.

**Reporting Method:**

This synthesis uses the publication standards for realist synthesis (RAMESES 1).

**Trial Registration:**

PROSPERO: 2024 CRD42024496072

**Summary**

- What is already known
  ○ Refusals of care can be distressing for both people with dementia and their caregivers.
  ○ There is some evidence that music, bathing techniques and communication strategies can reduce refusals of care from people with dementia.
- What this paper adds
  ○ Explanatory theories describing caregiver‐created contexts and causal mechanisms of programme features where caregivers can improve personal care interactions for people with dementia, which could increase positive outcomes.
  ○ Insight into how caregiver actions work to reduce refusals of care from people with dementia and in what circumstances.
  ○ Key mechanisms underlying acceptance of care by people with dementia include feelings of trust and safety, a connection with caregivers, working together, care feeling manageable and a sense of control.
- Implications for practice and policy
  ○ Our findings provide theoretical knowledge and practical care strategies that could be helpful for those directly assisting people with dementia or working to improve personal care practices for people with dementia such as nurses and paid and unpaid caregivers.

## 1 Background

People with moderate to advanced dementia develop considerable needs for assistance with their personal care (Raimo et al. 2024; Alzheimer's Disease International 2023). Assistance with personal care includes help with basic activities of daily living, such as washing, dressing, eating and going to the toilet (Prizer and Zimmerman 2018). Often, family caregivers provide such assistance (Amato et al. 2021). As more support is required, paid caregivers are frequently brought in to help within the home or the person with dementia may move into a care home (Beerens et al. 2014). The term ‘caregivers’ refers to unpaid and paid caregivers of people with dementia, including nurses, in any setting.

Personal care is a key opportunity for engagement (Haunch et al. 2023); however, sometimes a person with dementia may decline assistance and refuse care. Refusals of care, also sometimes termed care‐resistant behaviour (e.g., Bratches et al. 2025), resistance‐to‐care (e.g., Gjellestad et al. 2022) or rejection of care (e.g., Shaw et al. 2023), are common in the moderate and advanced stages of dementia (Bratches et al. 2025; Backhouse, Killett, et al. 2022). Refusals include the person with dementia moving away, clamping their jaw, stiffening their body or becoming angry (Backhouse, Khondoker, et al. 2022). Refusals can be caused by various factors such as the caregiver approach, for example if caregivers do not listen to the person (Backhouse, Jeon, et al. 2024) or act too quickly. The person with dementia may not recognise the caregiver or understand their intentions (Galindo‐Garre et al. 2015; Volicer 2021); they may have unmet needs such as being hungry, thirsty, in pain or uncomfortable (Ishii et al. 2012; Shaw et al. 2022), have high dependency in activities of daily living, be distressed (Backhouse, Killett, et al. 2022) or be experiencing depression or psychotic symptoms such as delusions or hallucinations (Ishii et al. 2010; Galindo‐Garre et al. 2015).

Refusals of care can be distressing for both the person with dementia (Featherstone et al. 2019) and their caregiver/s, contributing to caregiver overload (Fauth et al. 2016). Refusals can also lead to negative consequences such as poor hygiene, loss of skin integrity (McGraw 2019) or in some cases, the use of medications or controlled restraint to enable care (Featherstone et al. 2019), or to hospitalisation, the person moving into a long‐term care setting or a psychiatric inpatient service (Krolak‐Salmon et al. 2016; Johnson et al. 2013).

Due to these consequences, refusals of care are important to address. There is some evidence that playing recorded music, singing and using different bathing techniques such as a towel bath or thermal bath can reduce refusals of care. Regarding communication, elderspeak (simplified, overly endearing and patronising communication) and controlling communication are associated with increased refusals and clear concise instructions with reducing them (Backhouse et al. 2020; Konno et al. 2014, 2024). However, little is known about which caregiver actions may help make personal care assistance more acceptable to a person with dementia and how successful interventions work. Therefore, the aim of this realist synthesis was to identify strategies and mechanisms of interventions between caregivers and people with dementia that contribute to reducing refusals of care and determine how they work in which contexts, why and for whom.

## 2 Methods

### 2.1 Research Design

This synthesis has drawn on the publication standards for realist synthesis (RAMESES 1) (Wong et al. 2014). The published protocol for this realist synthesis outlines methods in depth (Backhouse, Killett, et al. 2024).

### 2.2 Rationale for Using Realist Synthesis

Realist synthesis was chosen for enabling examination of intervention components or strategies accounting for context, mechanisms and outcomes, providing evidence‐based theories, referred to as programmes and pragmatic conclusions to explain generative causation (Rycroft‐Malone et al. 2012; Jagosh 2019). Therefore, a realist synthesis could enable the generation of ideas (programme theories) to understand why a person with dementia would be more likely to accept personal care assistance and use evidence from sources and interviews to refine those ideas. In realist research, context refers to aspects such as the conditions or interrelationships that trigger causal mechanisms; mechanisms are the resources and hidden psychosocial underlying responses of relevant programme participants (here, caregivers and people with dementia) and outcomes refer to the conditions influenced by the causal mechanisms (De Brún and McAuliffe 2020; de Weger et al. 2020). This realist synthesis draws on Pawson et al.'s (2005) key steps in realist review and has three stages: (1) initial programme theory development and prioritisation, (2) literature search, review and synthesis of evidence to refine the programme theories and (3) further refinement of evidence‐based programme theories, using data from stakeholder interviews and team meetings.

### 2.3 Initial Programme Theory Development

Initial programme theories (IPTs) were generated through three activities: (1) assessing interview transcripts from family carers, care home staff (Backhouse et al. 2022) and people with dementia (Backhouse et al. 2025), and video‐recorded personal care interactions from a previous study conducted by the first author (Backhouse, Jeon, et al. 2024). Video‐recorded observations were of activities where the person with dementia had some clothes on, such as hair washing, oral care and nail cutting. Interactions were between family caregivers and people with dementia supported at home and care home staff and residents with dementia; (2) scoping the literature and (3) team discussions. If (a), Then (b), Because (c) statements were created and refined through grouping similar ideas from the data and key literature and team discussions (see study protocol for details of how the IPTs were generated and refined; Backhouse, Killett, et al. 2024). Although some IPTs covered caregiver mechanisms, to make the scope of the synthesis manageable, a decision was made to prioritise only those IPTs where mechanisms related to people with dementia.

In realist syntheses, mid‐range theories are looked for once IPTs are generated to help provide some broader explanation about the subject of interest. The mid‐range theory is different to the realist programme theories (findings) generated during the study; it is used to provide deeper thinking and aid interpretation. Self‐determination Theory was the mid‐range theory used to guide theory building (Ryan and Deci 2017, 2020). We chose this theory over other potential mid‐range theories for its explanatory properties because it fit best with our IPTs and the evidence we had looked at. In this way, the mid‐range theory choice was data driven.

### 2.4 Searching Processes

The main database searches were conducted on 22.04.2024 in OVID MEDLINE, OVID EMBASE, EBSCO PsycINFO, EBSCO CINAHL Ultimate, Cochrane CENTRAL register of controlled Trials and Web of Science. The search syntax covered three areas: dementia, personal care and refusals/acceptance (see Data S2). Limits on the searches were English language, humans and the years 2000 to April 2024. There were no limits on study methodology or type of resource/document. All citations were exported to Endnote x21 to remove duplicates before exporting the remaining citations to Excel.

Further searches were conducted iteratively via Google to explore relevant websites including interventions such as Teepasnow.com and Google Scholar for individual programme theories as the synthesis progressed. A further structured search focused on trust and safety was conducted to examine this overarching programme theory.

### 2.5 Selection and Appraisal of Documents

The first author screened all titles in Excel, removing any that were clearly not dementia related or out of scope for this study. Following title screening, the first author assessed all abstracts and full texts for inclusion. All other authors initially double‐screened a subset of titles and sources for inclusion to enhance validity and discrepancies were discussed at a team meeting until consensus was reached. Inclusion criteria had three features: (1) focus on people with dementia, (2) focus on personal care interactions in some way and (3) includes useful information for program theories regarding an intervention or strategy to reduce refusals of care or improve personal care for people with dementia. We excluded sources not focused on personal care interactions between people with dementia and caregivers, or those focused on end‐of‐life care. We found limited sources from hospital, family and home‐care settings, so a quota system for source inclusion was not used.

### 2.6 Quality

Assessments of relevance, richness and rigour (Pawson et al. 2005; Dada et al. 2023) were conducted on all potential sources to assess quality and each source's ability to contribute to theory building. Relevance was assessed on whether the source contained data applicable to the topic area or programme theories, and richness on whether the source could contribute sufficiently to theory building or testing (Dada et al. 2023; Wong et al. 2013). Rigour was judged on the extent to which the method generating the insight was credible and trustworthy (Wong et al. 2013) and whether the theory was coherent (Dada et al. 2023). Resources with low relevance and richness were excluded; those with low rigour were included if they could sufficiently contribute to theory building.

### 2.7 Data Extraction

Extracted data included authors, title, journal, country of origin, publication date, care setting, sample size and composition including stage of dementia and caregiver type, methods, design, analysis, contextual factors, mechanisms resource, mechanisms responses, outcomes, evidence relating to initial programme theories, links to IPTs and quality assessments of relevance, richness and rigour. These categories were likely to provide the evidence we needed for source descriptions and programme development. Data were extracted by the first author, with a subset of sources initially double‐extracted by other authors, to support quality assurance.

### 2.8 Analysis and Synthesis Processes

Data characterising the included sources were collated in a table and vote counted. Prioritised IPTs were populated with relevant extracted data generally and if possible, under context‐mechanism‐outcome (CMO) configurations. Using Drawio (a diagramming and collating tool), each author grouped data around IPTs and new ideas. The IPTs were then revised during team discussions considering the data and their fit to theories to develop evidence‐based CMOs. Many caregiver (mechanism resource) actions were the same from multiple sources (see supporting information Data S1 for extracted actions of caregivers and the mechanism response/s they were linked to). Synthesis occurred through ongoing examination of each of the theories and relevant data considering causal insights. The first author re‐read extracted data focusing on the programme theories considering meanings, theories and establishing connections between concepts. Regular team meetings examined and interrogated developing theories and CMOs alongside the evidence.

No subcategory synthesis took place due to the nature of the extracted data being relevant for all settings; however, differences related to ‘for who’ the theories related to were considered.

### 2.9 Public Involvement

This synthesis has involved the Stevenage Dementia Involvement Group, the Lived Experience Advisory Group for the OPTIMISED DEMCARE study and public advisors regularly throughout. Representatives have provided advice on initial programme theories, evidence‐based programme theories and synthesised stakeholder evidence.

### 2.10 Interviews

To refine the programme theories, we used a qualitative descriptive approach to conduct 15 stakeholder interviews (see Table 2). We used purposive sampling to gain a wide range of stakeholders to test out our ideas with people with varied views and experiences. Interviews were semi‐structured and explored reactions to the evidence‐based programme theories to enhance validity (Pawson et al. 2005). Interviews adopted a teacher‐learning function (Pawson 1996), using the ‘I'll show‐you‐my‐theory‐if‐you'll‐show‐me‐yours’ (Pawson 1996, 307) process, which allowed theories to be introduced in a way that stakeholders could fill in missing parts and thereby contribute to falsify, confirm or refine programme theories. Some stakeholders required more information than others.

### 2.11 Interview Data Synthesis

Interview data were audio‐recorded, transcribed and collated under each evidenced programme theory for each participant in an Excel sheet. Collated data were assessed and integrated with existing evidence to refine programme theories. Trustworthiness was generated through clear documentation and data management strategies, triangulation with existing evidenced ideas, comprehensive explanations, consistent collation of data, public involvement in synthesis and discussion, peer debriefing and reflexivity in regular team meetings. Public involvement meetings and team discussions were held to make decisions about refinements.

### 2.12 Ethics

This research was conducted in line with the Declaration of Helsinki statement of ethical principles for medical research involving human participants. Participation was voluntary and all participants gave informed consent. The Social Care Research Ethics Committee gave a favourable opinion for the interview stage of this study (REC reference: 24/IEC08/0007; IRAS project ID: 338274; Date 08.04.2024).

## 3 Results

### 3.1 Search Outcomes and Included Sources

**FIGURE 1:**
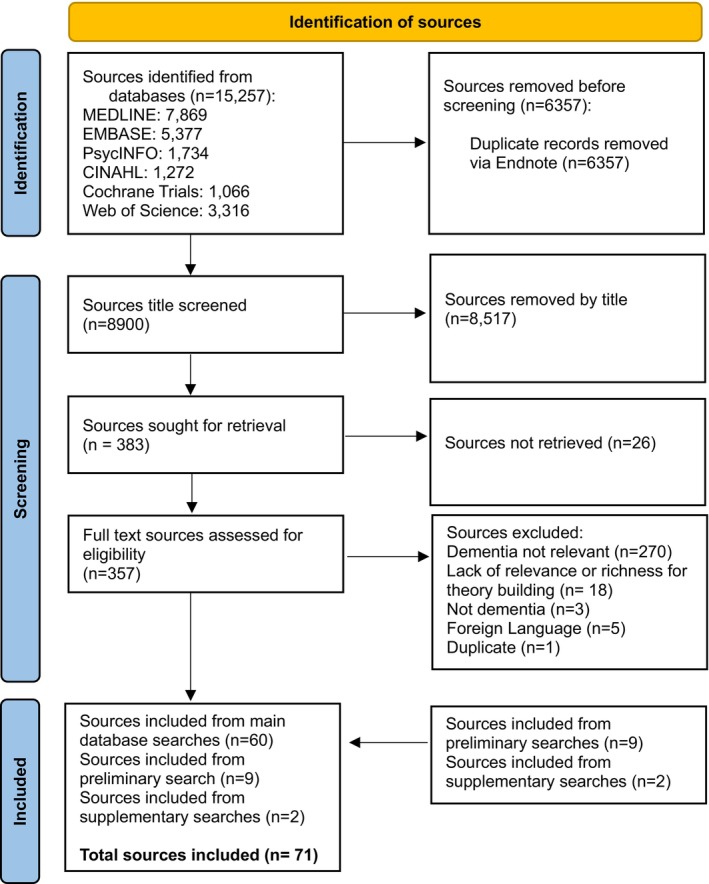
PRISMA flow diagram.

Our main search identified 15,257 sources (see Figure 1); after duplicates were removed, 8900 titles were screened and 357 full‐text sources were assessed. Including our preliminary searches and iterative searches, once screening and selection had taken place, 71 sources were included in the review (see Supporting Information Data S2 for reference list of included sources). Table 1 shows the characteristics of the included sources. Sources were from 22 countries, predominantly from the United States (21), the United Kingdom (11) and Sweden (5). Care settings were long‐term care settings (care homes/nursing homes) (44), family (7) and hospital settings (6), home care (3), day care (1), assisted living (1) and a dentist (1). Most sources covered generic approaches to all personal care activities (32), with others focusing on mealtimes (16), oral hygiene (9), dressing (8), morning care (6), continence care (5), showering or bathing (4), evening care (1) or medication administration (1). All sources were published articles except for two website sources. Sources included evidence of effectiveness (15), intervention descriptions (14), interviews (13), reviews (10), observations (7), statistical associations (5), discussion pieces (5), focus groups or workshops (5), case studies (2), document analysis (2) and surveys (2). Sample sizes varied from 0 to 4156 depending on the type of source and/or the methods used. Quality assessment results showed all sources high for relevance, 61 high and 11 medium for richness, and 6 high, 50 medium and 16 low for rigour.

**TABLE 1:** Source characteristics.

Reference and quality	Type of source and evidence	Country	Care setting: sample	Aim	Personal care activity
Amella (2002) Relevance—High Richness—High Rigour—Medium	– Published article – Evidence of effectiveness	USA	Nursing home: 53 Certified nursing assistants, 53 people with late‐stage dementia	To determine if resistance or willingness to accept assistance at meals by persons with dementia could be predicted by various personal interaction and contextual factors	Mealtimes
Apesoa‐Varano (2020) Relevance—High Richness—High Rigour—High	– Published article – Interviews	USA	Family homes: 15 family caregivers	To advance a feminist/labor process perspective by theorising it as valuable and meaningful activity carried out at the juncture of illness and the home	All care activities
Ashida et al. (2024) Relevance—High Richness—High Rigour—Medium	– Published article – Intervention description	USA	Family and paid caregivers: 16 family caregivers, 15 paid caregivers (Certified nursing assistant, RN, home health aide)	To develop and pilot test an App to help family and paid caregivers perform high quality oral hygiene care	Oral hygiene
Astorga et al. (2023) Relevance—High Richness—High Rigour—Low	– Published article – Intervention description	USA and Mexico	Day care and nursing home: 6 professional caregivers—interview, 14 caregivers (12 informal, 2 formal)—survey	To design and evaluate a social robot aimed at assisting people with dementia with disruptive eating behaviours	Mealtimes
Backhouse et al. (2020) Relevance—High Richness—High Rigour—Medium	– Published article – Review	Not applicable	All settings: Not applicable	To identify possible strategies and interventions to reduce or cope with refusals of care in dementia and determine the evidence for these	All care activities
Backhouse, Killett, et al. (2024) Relevance—High Richness—High Rigour—Medium	– Published article – Observations	UK	Care homes and family homes: 14 people with advanced dementia and 12 caregivers (7 care‐home staff, 5 family caregivers)	To examine features of personal care interactions between caregivers and people with advanced dementia to understand how care may be improved	All care activities
Backhouse, Jeon, et al. (2022) Relevance—High Richness—High Rigour—Medium	– Published article – Interviews	UK	Care homes and family homes: 12 care assistants and 20 family caregivers	To examine caregivers' (care‐home staff and family caregivers) experiences of managing refusals of personal care in advanced dementia	All care activities
Backhouse and Ruston (2022) Relevance—High Richness—High Rigour—Medium	– Published article – Interviews	UK	Home care: 17 homecare workers	To explore the experiences of home‐care workers and the knowledge and skills they rely on when providing personal care to people with dementia	All care activities
Boersma et al. (2017) Relevance—High Richness—High Rigour—Medium	– Published article – Intervention description	The Netherlands	Nursing home wards: 42 nursing home caregivers, 12 stakeholders	To perform a process analysis of the implementation of the Veder contact method for gaining insight into factors that influence successful implementation	All care activities
Bray et al. (2021) Relevance—High Richness—High Rigour—Medium	– Published article – Review	Not applicable	Care homes: Not applicable	To assess the quality of research evidence for the different activity components for the psycho‐social Namaste Care intervention for care home residents with advanced dementia	Mealtimes/morning care
Buse and Twigg (2018) Relevance—High Richness—High Rigour—High	– Published article – Interviews, observations, case studies	UK	Care homes and family homes: 32 case studies of people with dementia; 29 family caregivers, 28 care home staff	To undertake a detailed examination of the act of dressing in the context of dementia and institutional care	Dressing
Cartwright et al. (2022) Relevance—High Richness—High Rigour—Medium	– Published article – Evidence of effectiveness	Australia	Memory support unit: Care staff and residents—No clear numbers specified	To examine the impact of a Montessori mealtime intervention for people with dementia to support the mealtime experience of residents and mealtime care practices of staff in a memory support unit	Mealtimes
Chang and Roberts (2008) Relevance—High Richness—High Rigour—Medium	– Published article – Interviews, observations	Taiwan	Nursing Home: 48 residents and 31 nursing assistants	To investigate factors related to feeding difficulty that is shown in the interaction between nursing assistants and elderly residents with dementia	Mealtimes
Chou et al. (2016) Relevance—High Richness—Medium Rigour—Low	– Published article – Evidence of effectiveness	Not stated	Nursing home: 4 residents with dementia	To reduce resistance‐to‐care and combative behaviours in nursing home residents with dementia by eliciting their positive affect	Dressing/continence care
Davidson (2007) Relevance—High Richness—High Rigour—Low	– Published article – Discussion	Not applicable	Not applicable: Not applicable	To present a simple framework to help build understanding as well as a systematic approach to dealing with resistance to care	All care activities
Del and Palace (2016) Relevance—High Richness—High Rigour—Medium	– Published article – Evidence of effectiveness	USA	Nursing home: 10 residents with dementia	To evaluate the piloting of the ‘good morning Mum and Dad’ non‐pharmacological intervention in addressing the challenges of providing care to cognitively impaired patients	Morning care
The Eden Alternative, UK (2025) Relevance—High Richness—High Rigour—Low	– Website – Intervention description	UK	Care homes	Not applicable: Eden alternative training	All care activities
Faraday et al. (2021) Relevance—High Richness—High Rigour—Medium	– Published article – Review	Not applicable	Care homes: Not applicable	To identify good practice in mealtime care for people with dementia living in care homes, by focusing on caregiver‐resident interactions at mealtimes	Mealtimes
Galindo‐Garre et al. (2015) Relevance—High Richness—Medium Rigour—Medium	– Published article – Statistical association	The Netherlands	Nursing homes and residential homes: 1101 residents with dementia	To analyse factors related to rejection of care and behaviours directed towards others in nursing home residents with dementia	All care activities
Gaugler et al. (2016) Relevance—High Richness—Medium Rigour—Medium	– Published article – Intervention description	USA	Nursing homes and assisted living facilities: 40 direct care workers	To determine whether direct care workers' knowledge of responding to dementia‐related behaviour increased following participation in the CARES Dementia‐Related Behaviour Online Training Program and if it was acceptable and useful	All care activities
Giang et al. (2023) Relevance—High Richness—High Rigour—Medium	– Published article – Intervention description	Singapore	Not applicable	This study aimed to comprehensively review the research literature to provide an overview of the effects of Humanitude on people with dementia and their caregivers	All care activities
Gilmore‐Bykovskyi (2015) Relevance—High Richness—High Rigour—Medium	– Published article – Observations	USA	Nursing home memory care units: 9 residents with dementia and 6 care staff	To develop procedures for collecting and coding sequential data from naturally occurring caregiver‐resident mealtime interactions	Mealtimes
Gjellestad et al. (2022) Relevance—High Richness—High Rigour—Medium	– Published article – Focus groups, interviews	Norway	Home health care: 18 nurses from home health care	To get insight into how nurses experience resistance to care from home‐dwelling persons with dementia	All care activities
Gjellestad et al. (2023) Relevance—High Richness—High Rigour—Low	– Published article – Document/care record analysis	Norway	Family home: 88 documents of forced treatment and care for home‐dwelling persons with dementia	To explore the use of trust‐building interventions in home‐dwelling persons with dementia resisting care, as described by health professionals in documents of decisions of forced treatment and care	All care activities
Graneheim et al. (2005) Relevance—High Richness—High Rigour—Low	– Published article – Interviews	Sweden	Residential home: 6 care providers (registered nurse or enrolled nurse)	To illuminate the meaning of interaction with people suffering from dementia and behavioural disturbances	All care activities
Gutman, Karbakhsh, et al. (2021) Relevance—High Richness—High Rigour—Low	– Published article – Evidence of effectiveness	Canada	Care homes: 8 long term care home residents	To determine outcome trends of exposure to Mindful Garden on number and type of BPSD exhibited by residents with dementia during bathing and on staff time to undress, shower and re‐dress them	Shower/dressing
Gutman, Vashisht, et al. (2021), Relevance—High Richness—Medium Rigour—Medium	– Published article – Evidence of effectiveness	Canada	Care homes: 13 long‐term care residents with dementia	To determine outcome trends of exposure to Mindful Garden on BPSD and duration of care during morning and evening care, two activities well recognised as problematic for people with dementia and caregivers	Morning care/evening care
Hammar, Emami, Engstrom, and Gotell (2011) Relevance—High Richness—High Rigour—Medium	– Published article – Evidence of effectiveness	Sweden	Nursing homes: 10 people with dementia and 10 caregivers	To describe how people with dementia and their caregivers express verbal and nonverbal communication and make eye contact during the care activity ‘getting dressed’, during morning care situations without and with music therapeutic caregiving	Morning care/dressing
Hammar, Emami, Gotell, and Engstrom (2011) Relevance—High Richness—High Rigour—Medium	– Published article – Evidence of effectiveness	Sweden	Nursing homes: 12 people with dementia and 10 professional caregivers	To describe people with dementias' expressions of resistiveness to care and expressions of emotions, while being cared for by their caregivers during morning care situations without and with music therapeutic caregiving	Morning care
Hanson et al. (2023) Relevance—High Richness—High Rigour—Low	– Published article – Intervention description	USA	Nursing homes: 146 nursing home staff	To facilitate dissemination, we developed ComfortFirst, a web‐based training toolkit with video demonstration of Comfort Matters practices	All care activities
Henriques et al. (2019) Relevance—High Richness—High Rigour—Low	– Published article – Intervention description	Portugal	Continuing care unit: 34 health professionals	To evaluate the contribution of the implementation of the Humanitude Care Methodology to the quality of health care in a Continuing Care Unit	All care activities
Ishii et al. (2010) Relevance—High Richness—Medium Rigour—Medium	– Published article – Statistical association	USA	Nursing homes: 3230 Nursing home residents	To identify the potentially modifiable resident‐level factors associated with rejection of care in nursing home (NH) residents	All care activities
Ishii et al. (2012) Relevance—High Richness—Medium Rigour—Medium	– Published article – Review	Not applicable	Not applicable	To propose a definition of rejection of care and develop a conceptual framework of rejection of care	All care activities
Jablonski et al. (2018) Relevance—High Richness—High Rigour—High	– Published article – Evidence of effectiveness	USA	Nursing homes: 101 nursing home residents with dementia	To test the efficacy of MOUTh (Managing Oral Hygiene Using Threat Reduction), a nonpharmacologic, relationship‐based intervention vs. control for nursing home (NH) residents with dementia who resisted mouth care	Oral hygiene
Jablonski, Therrien, and Kolanowski (2011) Relevance—High Richness—High Rigour—Medium	– Published article – Discussion	Not applicable	Not applicable	To describe how the neurobiological principles of threat perception and fear response can support clinical approaches to prevent and reduce care‐resistant behaviours during mouth care	Oral hygiene
Jablonski, Therrien, Mahoney, et al. (2011) Relevance—High Richness—High Rigour—Medium	– Published article – Evidence of effectiveness	USA	Nursing home: 7 residents with dementia	To test the feasibility of an intervention designed to reduce care‐resistant behaviours in persons with moderate‐to‐severe dementia during oral hygiene activities	Oral hygiene
Jablonski‐Jaudon et al. (2016) Relevance—High Richness—High Rigour—Low	– Published article – Intervention description	USA	Not applicable	To describe a personalised practice originally conceived as a way to prevent and minimise care‐resistant behaviour to provide mouth care to older adult with dementia	Oral hygiene
James et al. (2020) Relevance—High Richness—High Rigour—Medium	– Published article – Intervention description	UK	Not applicable	To provide guidance on the management of challenging behaviours (CBs) in dementia care, and introduce concepts from positive behavioural support not usually applied to dementia	All care activities
Jensen et al. (2023) Relevance—High Richness—Medium Rigour—Medium	– Published article – Review	Not applicable	Orthopaedic hospital: Not applicable	To collect what is known about the care of people with dementia when they require a hospital admission for an orthopaedic surgical procedure	All care activities
Jung et al. (2024) Relevance—High Richness—High Rigour—Medium	– Published article – Observations	Korea	Long‐term care facilities: 10 residents and 24 staff members	To explore the mealtime difficulties faced by residents with dementia in long‐term care facilities in Korea	Mealtimes
Kobayashi et al. (2021) Relevance—High Richness—Medium Rigour—Medium	– Published article – Intervention description	Japan	Dentist practices: 26 dentists and 19 dental hygienists	To assess whether a multimodal comprehensive care methodology training programme, Humanitude, was associated with an improvement in empathy for people with dementia among oral health care professionals	Oral hygiene
Konno et al. (2014) Relevance—High Richness—High Rigour—Medium	– Published article – Review	Not applicable	Nursing homes: Not applicable	To determine the effectiveness of interventions intended to reduce the frequency and intensity of resistance‐to‐care behaviours during assisted personal‐care activities	All care activities
Konno et al. (2024) Relevance—High Richness—High Rigour—High	– Published article – Review	Not applicable	Not applicable	To determine the best strategies for assisted bathing or showering for older adults with dementia	Bathing, showering
Kristensen and Peoples (2020) Relevance—High Richness—Medium Rigour—Medium	– Published article – Review	Not applicable	Institutional settings	To investigate experiences related to quality of life in people with dementia living in institutional settings	All care activities
Kutsumi et al. (2009) Relevance – High Richness—High Rigour—Medium	– Published article – Interviews, survey	Japan	Long‐term care facilities: Interviews: 15 care providers; Survey: 275 care providers	To examine techniques for managing BPSD within long‐term care facilities	All care activities
Langley et al. (2022) Relevance—High Richness—High Rigour—Medium	– Published article – Workshops	UK	Care homes: 9‐to 18 for each workshop	To (1) Explore the challenges of providing daily oral care in care homes; (2) understand oral care practices provided by care home staff; (3) co‐design practical resources supporting care home staff in these activities.	Oral hygiene
Levy‐Storms et al. (2016) Relevance—High Richness—High Rigour—Medium	– Published article – Evidence of effectiveness	USA	Dementia Care Unit in a nursing home: 16 certified nursing assistants	To evaluate the effectiveness of a therapeutic communication‐training program, designed for certified nursing assistants caring for nursing home residents with dementia	Mealtimes
Luk et al. (2017) Relevance—High Richness—High Rigour—Low	– Published article – Discussion	China	Not applicable	To discuss the reliance on tube feeding in advanced dementia	Mealtimes
Mahoney et al. (2016) Relevance—High Richness—High Rigour—Medium	– Published article – Focus groups	USA	Community: 20 family caregivers	To gain an understanding of Latino/Hispanic caregivers' dementia‐related dressing issues, their impressions of using a ‘smart’ context‐aware dresser to coach dressing, and recommendations to improve its acceptability.	Dressing
Moniz‐Cook et al. (2003) Relevance—High Richness—High Rigour—Medium	– Published article – Case studies	UK	Nursing home: 5 residents with dementia	To describe psychosocial interventions in five people, who presented with uncooperative and difficult behaviour at mealtimes and during assistance with self‐care tasks	Mealtimes/self‐care
Nagahama et al. (2022) Relevance—High Richness—High Rigour—Low	– Published article – Document analysis	Japan	Hospital: 29 patients with food refusal on admission	To evaluate the outcome of interventions against food refusal in patients who were admitted to our hospital	Mealtimes
O'Brien et al. (2020) Relevance—High Richness—High Rigour—Medium	– Published article – Observations	UK	Acute hospital wards: Nurses 19; Allied health professionals 11; Doctors 11; Total 41	To investigate recurring interactional difficulties around HCP requests to carry out health and social care tasks and subsequent reluctance or refusal on the part of people with dementia	All care activities
O'Connor et al. 2011 Relevance—High Richness—Medium Rigour—Low	‐Published article ‐Evidence of effectiveness	Australia	Nursing home: 1 resident	To investigate the effect of Video Simulated Presence for decreasing resistance to care and increasing participation in activities of daily living to improve basic care of people with dementia	Mealtimes and medication
Ostaszkiewicz, Dunning, and Dickson‐Swift (2020) Relevance—High Richness—Medium Rigour—Medium	– Published article – Intervention description	Australia	Care homes: Not applicable	To support aged care providers to translate rights, principles, standards, and recommendations into practice	Continence care
Ostaszkiewicz, Dickson‐Swift, et al. (2020) Relevance—High Richness—High Rigour—Medium	– Published article – Review	Not applicable	Long‐term care settings	To explore, describe and explain the concept of dignity as it relates to continence care for older people requiring long‐term care	Continence care
Prizer and Zimmerman (2018) Relevance—High Richness—High Rigour—Medium	– Published article – Review	Not applicable	Not applicable	To summarise the practices to care for early stage, middle stage, and late‐stage activity of daily living needs, and examines commonalities across activity of daily living needs and the extent to which practices are reflected in guidelines and/or evidence	Dressing, continence care, mealtimes
Rey et al. (2020) Relevance—High Richness—High Rigour—Low	– Published article – Discussion	Not applicable	Nursing settings	To describe and discuss how the fundamentals of care practice process can help nurses understand people with Alzheimer's disease who show resistance to care behaviours during bodily care	All care activities
Roberto et al. (2024) Relevance—High Richness—High Rigour—Medium	– Published article – Interviews	USA	Family/community dwelling: 30 family caregivers	To identify the ways caregivers managed behaviours and bar graphs to examine management approaches relative to categories of behaviours and caregiver demographic and emotional well‐being variables	All care activities
Shaw et al. (2023) Relevance—High Richness—High Rigour—Medium	– Published article – Statistical association	USA	Hospital: 16 patients with dementia, 53 nursing staff	To further understand the relationship between rejection of care and pain and delirium in hospitalised patients with dementia by identifying which rejection of care behaviours are associated with pain and delirium	All care activities
Shaw et al. (2022) Relevance—High Richness—High Rigour—Medium	– Published article – Statistical association	USA	Hospital: 16 patients with dementia, 53 nursing staff	To determining the impact of elderspeak communication by nursing staff on rejection of care by hospitalised people with dementia in acute care settings	All care activities
Sloane et al. (2004) Relevance—High Richness—High Rigour—High	– Published article – Evidence of effectiveness	USA	Skilled nursing facilities: 73 residents with agitation during bathing and 37 nursing assistants	To evaluate the efficacy of two nonpharmacological techniques in reducing agitation, aggression, and discomfort in nursing home residents with dementia	Showering, towel bathing
Sonde et al. (2011) Relevance—High Richness—High Rigour—Medium	– Published article – Focus groups, interviews	Sweden	Nursing home: 9 care providers (enrolled nurses/nursing assistants) focus groups; 4 nurse interviews	To describe care providers' perception of and reasoning for the oral care for nursing home residents with dementia and to describe registered nurses' reasoning in relation to their responsibility for monitoring oral care interventions within the regular caregiving routines for nursing home residents with dementia	Oral hygiene
Stanyon et al. (2019) Relevance—High Richness—High Rigour—Medium	‐Published article ‐Evidence of effectiveness	UK	Residential care setting: 3 dyads (person with dementia/care worker)	To determine whether varying the communication style of care assistants, encouraging them to use direct instructions and allowing more time for residents' responses influenced the communicative behaviour of care home residents with dementia	Morning care
Snow (2022) Relevance—High Richness—High Rigour—Low	– Website – Intervention description	USA	Not applicable	Not applicable: Positive approach to care	All care activities
Thorsen and Nielsen (2023) Relevance—High Richness—High Rigour—High	– Published article – Observations, interviews, focus groups	Denmark	Nursing homes: Numbers not described‐ Care assistants (nursing home and home care), nurses, consultants, relatives, 1 social worker, 1 lawyer	To suggest ways in which vocationally trained care assistants may understand and work with issues of (mis)trust in relation to people diagnosed with dementia and thus improve the quality of care within this field of work	All care activities
Tosato et al. (2012) Relevance—High Richness—High Rigour—Medium	– Published article – Statistical association	Czech Republic, England, Finland, France, Germany, Italy, The Netherlands, Israel	Nursing homes: 4156 residents	To assess the association of pain with behavioural and psychiatric symptoms in a population of nursing home residents with cognitive impairment in Europe	All care activities
Villar et al. (2022) Relevance—High Richness—High Rigour—Medium	– Published article – Interviews	Spain	Long‐term care facilities: 21 nursing assistants, 21 technical staff	To explore the perception of common and best practices for dealing with resistance to eating of persons with dementia living in long‐term care facilities	Mealtimes
Volicer (2021) Relevance—High Richness—High Rigour—Low	– Published article – Discussion	Not applicable	Not applicable	To discuss the difference between reactive and proactive aggression and how these can be managed	All care activities
Werner et al. (2002) Relevance—High Richness—High Rigour—Medium	– Published article – Survey	Isreal	Psychiatric hospitals and nursing homes: 34 nurses in psychiatric hospitals, 16 nurses in nursing homes	To examine differences in the use of interventions by nurses in different care settings to manage resistance to care with eating and dressing	Mealtimes/dressing
Westerberg and Strandberg (2007) Relevance—High Richness—High Rigour—Medium	– Published article – Interviews	Sweden	Residential homes: 12 nursing assistants	To explore whether it was possible to identify different sequences and themes in the descriptions of the shower situations that would enhance the understanding of the content and qualities of this kind of work	Shower
Zimmerman et al. (2014) Relevance—High Richness—High Rigour—Medium	– Published article – Intervention description	USA	Not applicable	To describe a new culture change practice, Mouth Care Without a Battle	Oral hygiene

### 3.2 Stakeholder and Interview Characteristics

Interviews took place with 15 stakeholders (see Table 2) including a person with dementia, family caregivers, home‐care workers, care home staff and professionals supporting caregivers. All were White British, 73% were female, and the average age was 58 years. Interviews lasted an average of 52 min, and most took place via online video call.

**TABLE 2:** Stakeholder and interview characteristics.

Stakeholders total *n* (%)	15
People with dementia	1 (6.7)
Family caregivers	4 (26.7)
Home care workers	3 (20)
Care home staff	3 (20)
Professionals supporting caregivers	4 (26.7)
Age average years (range)	58 (27–80)
Gender *n* (%)	
Male	4 (26.7)
Female	11 (73.3)
Ethnicity	
White British *n* (%)	15 (100)
Length of interview average minutes (range)	52.50 (29.31–66.12)
Mode of interview *n* (%)	
Face‐to‐face	3 (20)
Telephone	1 (6.7)
Online video call	11 (73.3)

### 3.3 Contexts, Mechanisms and Outcomes

**FIGURE 2:**
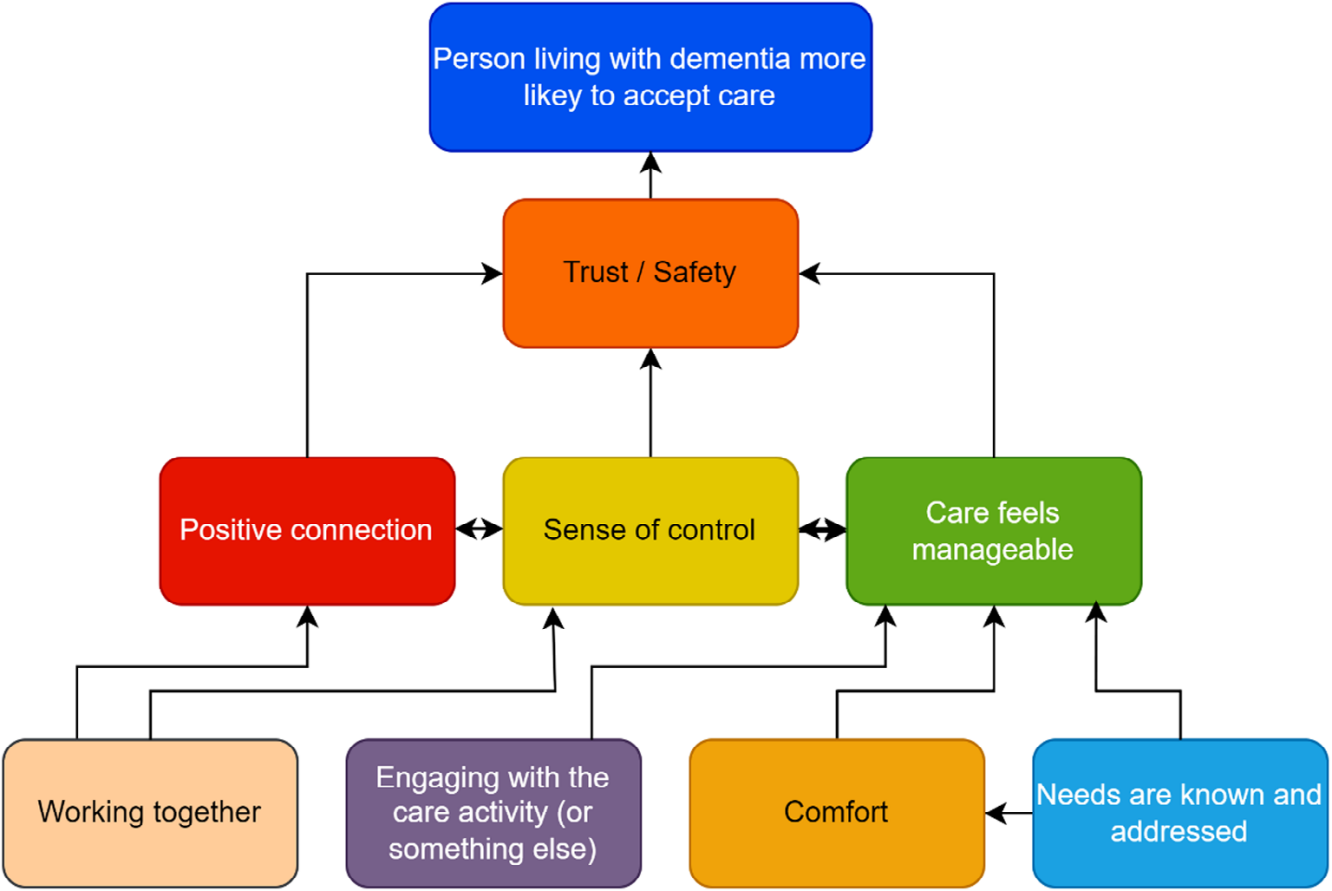
Model of mechanism responses from people living with dementia leading to higher likelihood of acceptance of personal care assistance.

The study generated eight evidenced and interrelated programme theories (Table 3). One overarching programme theory (trust and safety) was developed with three further programme theories (sense of control, positive connection, care feels manageable) feeding into the main theory and four other theories (working together, comfort, needs are addressed and known, engaging in the care activity (or something non‐care related)) building into those (see Figure 2). Our public advisers and stakeholder interviews endorsed all eight theories and provided nuances for each. Our programme theories focus closely on care interactions. It was beyond the scope of this study to examine broader contextual factors; for example, caregiver time availability; care cultures, training and support; caregiver past experiences and knowledge of dementia care.

**TABLE 3:** Programme theories.

Theory	Context	+	Mechanism		=	Outcome	Evidence
			Resource	Response			
Overarching theory Theory 1: Trust and Safety *All theories contribute to the trust and safety mechanism (see Figure 2)	When a caregiver creates a kind emotional environment, and is dependable, innocuous, and attentive	+	Through these strategies: – Learns about the person and adapts to their preferences – Builds a relationship with the person – Tells the person what they are doing – Stops when asked – Positions themselves non‐intimidatingly – Makes sure the person has something to hold on to when transferring/mobilising – Ensures physical safety – Reassures the person – Validates the person's experiences – Shows confidence in their actions – Says farewell at the end of care – Thanks the person after care	The person with dementia trusts the caregiver and feels safe in the interaction	=	And is more likely to accept assistance with personal care	Sources *n* = 26 Backhouse et al. (2020), Backhouse, Jeon, et al. (2024), Backhouse and Ruston (2022), Boersma et al. (2017), Buse and Twigg (2018), Galindo‐Garre et al. (2015), Gaugler et al. (2016), Giang et al. (2023), Gjellestad et al. (2022), Gjellestad et al. (2023), Henriques et al. (2019), James et al. (2020), Kobayashi et al. (2021), Kristensen and Peoples (2020), Kutsumi et al. (2009), Moniz‐Cook et al. (2003), O'Brien et al. (2020), O'Connor et al. (2011), Ostaszkiewicz, Dickson‐Swift, et al. (2020), Prizer and Zimmerman (2018), Roberto et al. (2024), Snow (2022), Sonde et al. (2011), Thorsen and Nielsen (2023), Volicer (2021), Zimmerman et al. (2014) Interviews *n* = 15
Theory 2: Sense of control	When a caregiver adopts a person‐led care approach and empowers the person	+	Through these strategies: – Learns about the person and adapts to their preferences – Offers choices (limited, with visual cues) – Establishes a joint goal – Shares control – Promotes independence/self‐care – Seeks consent/assent ‘do you mind if…’ – Lets the person lead – Tells the person what they are doing at each step to enable assent or refusal – Stops when asked – If the person is not ready, leave and return later	The person with dementia has a sense of being in control	=	And is more likely to accept assistance with personal care	Sources *n* = 44 Apesoa‐Varano (2020), Ashida et al. (2024), Backhouse, Jeon, et al. (2024), Backhouse, Jeon, et al. (2022), Backhouse and Ruston (2022), Buse and Twigg (2018), Cartwright et al. (2022), Chang and Roberts (2008), Davidson (2007), The Eden Alternative, UK (2025), Faraday et al. (2021), Galindo‐Garre et al. (2015), Gaugler et al. (2016), Giang et al. (2023), Gilmore‐Bykovskyi (2015), Gjellestad et al. (2023), Hanson et al. (2023), Henriques et al. (2019), Ishii et al. (2012), Jablonski et al. (2018), Jablonski, Therrien, and Kolanowski (2011), James et al. (2020), Jensen et al. (2023), Jung et al. (2024), Kobayashi et al. (2021), Konno et al. (2014), Konno et al. (2024), Kristensen and Peoples (2020), Langley et al. (2022), Moniz‐Cook et al. (2003), Nagahama et al. (2022), O'Brien et al. (2020), Ostaszkiewicz, Dunning, and Dickson‐Swift (2020), Ostaszkiewicz, Dickson‐Swift, et al. (2020), Prizer and Zimmerman (2018), Rey et al. (2020), Roberto et al. (2024), Sloane et al. (2004), Snow (2022), Sonde et al. (2011), Villar et al. (2022), Volicer (2021), Westerberg and Strandberg (2007), Zimmerman et al. (2014) Interviews *n* = 15
Theory 3: Positive connection	When a caregiver knows the person's history and/or values the person as a human and approaches the person respectfully and genuinely and creates a warm, encouraging and friendly social environment	+	Through these strategies: – Knocks on the door – Approaches from the front – Smiles – Makes eye contact, unless person is refusing – Greets/announces their arrival – Uses person's name – Uses a calm soft tone – Is friendly – Complements the person – Listens to the person – Uses relaxed body language – Is present in the moment – Uses appropriate touch – Uses known words – Plays music – Sings – Offers reassurance – Tells the person what is happening – Has fun together with the person – Uses humour (if appropriate) – Involves the person throughout – Makes a connection before mentioning care – Employs task and social chat – Uses or learns from ‘best’ relationships (familiar/preferred caregiver) – Involves the person's family – Shows confidence in their actions – Builds rapport – Shows they are a safe person – Enters the person's reality – Is empathetic – Validates the person—acknowledge and address issues	The person with dementia feels a sense of connection, belonging, inclusion and self‐worth	=	And is more likely to accept assistance with personal care	Sources *n* = 54 Apesoa‐Varano (2020), Ashida et al. (2024), Backhouse et al. (2020), Backhouse, Jeon, et al. (2024), Backhouse, Jeon, et al. (2022), Backhouse and Ruston (2022), Boersma et al. (2017), Bray et al. (2021), Buse and Twigg (2018), Cartwright et al. (2022), Chou et al. (2016), Davidson (2007), Del and Palace (2016), Faraday et al. (2021), Gaugler et al. (2016), Giang et al. (2023), Gilmore‐Bykovskyi (2015), Gjellestad et al. (2022), Gjellestad et al. (2023), Hammar, Emami, Engstrom, and Gotell (2011), Hammar, Emami, Gotell, and Engstrom (2011), Hanson et al. (2023), Henriques et al. (2019), Ishii et al. (2012), Jablonski et al. (2018), Jablonski, Therrien, and Kolanowski (2011), Jablonski, Therrien, Mahoney, et al. (2011), Jablonski‐Jaudon et al. (2016), James et al. (2020), Jensen et al. (2023), Jung et al. (2024), Kobayashi et al. (2021), Konno et al. (2014), Konno et al. (2024), Kristensen and Peoples (2020), Kutsumi et al. (2009), Langley et al. (2022), Levy‐Storms et al. (2016), Luk et al. (2017), Moniz‐Cook et al. (2003), O'Brien et al. (2020), O'Connor et al. (2011), Ostaszkiewicz, Dunning, and Dickson‐Swift (2020), Ostaszkiewicz, Dickson‐Swift, et al. (2020), Prizer and Zimmerman (2018), Rey et al. (2020), Roberto et al. (2024), Sloane et al. (2004), Snow (2022), Thorsen and Nielsen (2023), Villar et al. (2022), Volicer (2021), Werner et al. (2002), Westerberg and Strandberg (2007), Zimmerman et al. (2014) Interviews *n* = 15
Theory 4: Care feels manageable	When the caregiver responds to the person in the moment and/or simplifies the sound of, or processes of, the care activity	+	Through these strategies: – Is flexible and adapts to the person each interaction – Guides the person through the activity in small steps – Builds the person's confidence – Employs person‐led care – Minimises the sound of care (‘just’ ‘a quick wash’) – Offers care assistance at a preferred time – Follows the person's usual routine – Uses known terminology – Offers a different mode of care (strip wash rather than shower) – Makes sure the person is in an appropriate position – Changes caregivers or brings in an additional caregiver – Offers limited information or choices – Uses an unhurried approach going at the person's own pace (unless the person is walking away or distressed due to cold) – Allows time for the person to self‐care	The person with dementia feels more capable of managing the care activity	=	And is more likely to accept assistance with personal care	Sources *n* = 43 Amella (2002), Backhouse, Jeon, et al. (2024), Backhouse, Jeon, et al. (2022), Backhouse and Ruston (2022), Buse and Twigg (2018), Cartwright et al. (2022), Chang and Roberts (2008), Davidson (2007), The Eden Alternative, UK (2025), Faraday et al. (2021), Gaugler et al. (2016), Gilmore‐Bykovskyi (2015), Gjellestad et al. (2022), Gjellestad et al. (2023), Graneheim et al. (2005), Hammar, Emami, Engstrom, and Gotell (2011), Jablonski et al. (2018), Jablonski, Therrien, and Kolanowski (2011), Jablonski, Therrien, Mahoney, et al. (2011), James et al. (2020), Jensen et al. (2023), Jung et al. (2024), Kobayashi et al. (2021), Konno et al. (2014), Kutsumi et al. (2009), Langley et al. (2022), Luk et al. (2017), Mahoney et al. (2016), Moniz‐Cook et al. (2003), O'Brien et al. (2020), Ostaszkiewicz, Dickson‐Swift, et al. (2020), Prizer and Zimmerman (2018), Rey et al. (2020), Sloane et al. (2004), Snow (2022), Sonde et al. (2011), Stanyon et al. (2019), Thorsen and Nielsen (2023), Villar et al. (2022), Volicer (2021), Werner et al. (2002), Westerberg and Strandberg (2007), Zimmerman et al. (2014) Interviews *n* = 14
Theory 5: Working together	When a caregiver views the person as a partner in the care interaction, and works together with them	+	Through these strategies: – Starts an activity and hands it over for the person to complete – Establishes a shared goal – Tells the person what they are doing – Gives the person a care item to hold – Uses the guiding hand technique – Involves the person and works with them – Uses inclusive and inviting terms such as ‘shall we?’ and ‘can you help me?’ ‘let's’	The person with dementia feels part of a collaborative activity, (a shared enterprise) generating a sense of achievement	=	And is more likely to accept assistance with personal care	Sources *n* = 20 Backhouse, Jeon, et al. (2024), Backhouse and Ruston (2022), Cartwright et al. (2022), Davidson (2007), Gjellestad et al. (2023), Jablonski et al. (2018), Jablonski, Therrien, and Kolanowski (2011), Jablonski, Therrien, Mahoney, et al. (2011), Jablonski‐Jaudon et al. (2016), James et al. (2020), Jung et al. (2024), Langley et al. (2022), O'Brien et al. (2020), Ostaszkiewicz, Dunning, and Dickson‐Swift (2020), Ostaszkiewicz, Dickson‐Swift, et al. (2020), Prizer and Zimmerman (2018), Snow (2022), Thorsen and Nielsen (2023), Westerberg and Strandberg (2007), Zimmerman et al. (2014) Interviews *n* = 14
Theory 6: Comfort	When the caregiver acts in a way that prioritises the person's experience of care	+	Through using these strategies: *Emotional comfort* – Connects with the person – Reassures the person – Positions themselves innocuously – Praises the person – Makes care enjoyable – Adapts to the person's preferences – Does not use elderspeak – Goes along with the person's reality – Validates the person – Leaves and try again later *Sensory comfort* – Employs music/singing – Uses touch, holds hands, strokes hair as appropriate to provide pleasurable care – Adapts the environment (lights, noise, busyness) *Physical comfort* – Addresses pain before care – Is gentle (e.g., with touch, water spray, toothbrush) – Ensures appropriate temperature/s (room, water, person)	The person with dementia feels increased comfort (emotional, sensory, physical)	=	And is more likely to accept assistance with personal care	Sources *n* = 49 Apesoa‐Varano (2020), Ashida et al. (2024), Astorga et al. (2023), Backhouse et al. (2020), Backhouse, Jeon, et al. (2024), Backhouse and Ruston (2022), Boersma et al. (2017), Bray et al. (2021), Buse and Twigg (2018), Davidson (2007), Del and Palace (2016), The Eden Alternative, UK (2025), Gaugler et al. (2016), Giang et al. (2023), Hammar, Emami, Gotell, and Engstrom (2011), Hanson et al. (2023), Henriques et al. (2019), Ishii et al. (2010), Ishii et al. (2012), Jablonski et al. (2018), Jablonski, Therrien, and Kolanowski (2011), Jablonski, Therrien, Mahoney, et al. (2011), James et al. (2020), Kobayashi et al. (2021), Konno et al. (2014), Konno et al. (2024), Kristensen and Peoples (2020), Kutsumi et al. (2009), Langley et al. (2022), Mahoney et al. (2016), Moniz‐Cook et al. (2003), Nagahama et al. (2022), O'Connor et al. (2011), Ostaszkiewicz, Dunning, and Dickson‐Swift (2020), Ostaszkiewicz, Dickson‐Swift, et al. (2020), Prizer and Zimmerman (2018), Rey et al. (2020), Roberto et al. (2024), Shaw et al. (2023), Shaw et al. (2022), Sloane et al. (2004), Snow (2022), Sonde et al. (2011), Thorsen and Nielsen (2023), Tosato et al. (2012), Villar et al. (2022), Volicer (2021), Westerberg and Strandberg (2007), Zimmerman et al. (2014) Interviews *n* = 15
Theory 7: Needs are known and addressed	When the caregiver assesses the person for cues and clues of unmet needs and acts to address them before introducing or starting care	+	Through these strategies: – Finds out about the person and how they are at that moment – Makes sure sensory supports are in place (hearing aid, glasses, teeth) – Addresses any physical discomfort before introducing or starting care – Resolves emotional distress before introducing or starting care – Ensures privacy and dignity – Considers and tries to address symptoms (pain, depression, delusions)	The person with dementia has a sense of peace and reassurance where their needs are known and met	=	And is more likely to accept assistance with personal care	Sources *n* = 21 Backhouse, Jeon, et al. (2022), Davidson (2007), Galindo‐Garre et al. (2015), Hanson et al. (2023), Ishii et al. (2010), Ishii et al. (2012), Jablonski et al. (2018), Jensen et al. (2023), Konno et al. (2014), Moniz‐Cook et al. (2003), Ostaszkiewicz, Dunning, and Dickson‐Swift (2020), Ostaszkiewicz, Dickson‐Swift, et al. (2020), Prizer and Zimmerman (2018), Rey et al. (2020), Shaw et al. (2023), Shaw et al. (2022), Sloane et al. (2004), Snow (2022), Tosato et al. (2012), Villar et al. (2022), Zimmerman et al. (2014) Interviews *n* = 15
Theory 8: Engaging with the care activity (or something else)	When the caregiver helps to focus the person's attention on to the care activity (or on something non‐care related)	+	Through these strategies: *To promote engagement with the care activity* – Explains what is going to happen next and waits for person to digest this – Uses clear, short instructions – Takes time – Repeats self if needed – Simplifies care – Explains why care is needed – Sits the person up (if able) – Removes distractions – Promotes self‐care – Validates and acknowledges the person's experience – Uses non‐verbal – Communication (gestures, demonstrate, guiding, touch) – Promotes contact with care objects *To motivate* – Encourages – Prompts – Praises – Enters the person's reality – Offers or points out a reward after care *To promote engagement with something else* – Gives the person something to hold (e.g., a comfort item) – Uses music/singing – Talks about something else during care	The person with dementia is engaged with what is happening during the care activity (or with something else if care can be distressing or unsettling)	=	And is more likely to accept assistance with personal care	Sources *n* = 48 Apesoa‐Varano (2020), Astorga et al. (2023), Backhouse et al. (2020), Backhouse, Jeon, et al. (2024), Backhouse, Jeon, et al. (2022), Backhouse and Ruston (2022), Boersma et al. (2017), Bray et al. (2021), Buse and Twigg (2018), Cartwright et al. (2022), Chang and Roberts (2008), Chou et al. (2016), Davidson (2007), Faraday et al. (2021), Galindo‐Garre et al. (2015), Giang et al. (2023), Gjellestad et al. (2023), Gutman, Vashisht, et al. (2021), Gutman, Karbakhsh, et al. (2021), Henriques et al. (2019), Jablonski et al. (2018), Jablonski, Therrien, and Kolanowski (2011), Jablonski, Therrien, Mahoney, et al. (2011), Jablonski‐Jaudon et al. (2016), James et al. (2020), Jensen et al. (2023), Jung et al. (2024), Konno et al. (2014), Konno et al. (2024), Kutsumi et al. (2009), Langley et al. (2022), Levy‐Storms et al. (2016), Luk et al. (2017), Mahoney et al. (2016), Moniz‐Cook et al. (2003), O'Brien et al. (2020), Prizer and Zimmerman (2018), Rey et al. (2020), Roberto et al. (2024), Sloane et al. (2004), Snow (2022), Sonde et al. (2011), Stanyon et al. (2019), Thorsen and Nielsen (2023), Volicer (2021), Werner et al. (2002), Westerberg and Strandberg (2007), Zimmerman et al. (2014) Interviews *n* = 14

*Note:* The person: the person with dementia.

#### 3.3.1 Trust and Safety

**When a caregiver creates a kind emotional environment, and is dependable, innocuous and attentive (Context, C), the person with dementia trusts the caregiver and feels safe in the interaction (Mechanism, M) and is more likely to accept assistance with personal care (Outcome, O)**.

The overarching programme theory related to the mechanisms of trust and safety was supported by 26 sources and 15 interviews. To improve care practice, data suggested that a person with dementia would be more likely to accept assistance with personal care if they trusted the caregiver and felt safe physically and emotionally (Table 3), those providing care should try to build trust and make the person feel safe. A family caregiver stated:

If they [person with dementia] don't have trust, then yes, then they will be fearful, they will feel insecure. (Family caregiver, spouse, female 2)

Thorsen and Nielsen (2023) found that caregivers needed to set the tone of care interactions to build trust, foregrounding social connections and integrating social and task aspects of care. Similarly, Gjellestad et al. (2023) concluded that ‘balancing safe care with the person's integrity’ was key for trust building in dementia care. Stakeholders agreed:

It's just finding that way of letting somebody know that you're safe. Somebody they can trust because it's such an intimate thing, isn't it? To have personal care …That that kind of connection and feeling that they know the person that they can trust them is really important (Care home staff, female, 4)

As well as all other programme theories feeding in to this programme theory, strategies caregivers could employ to directly enact this mechanism included: learning about the person and adapting to their preferences, building a relationship with the person, telling the person what they are doing, stopping when asked, making sure the person has something to hold on to when mobilising, ensuring physical safety, reassuring the person, validating the person's experiences, showing confidence in their own actions, saying farewell at the end of care and thanking the person after the interaction.

Scheduling the next care interaction was reported in the literature to generate trust (Henriques et al. 2019; Kobayashi et al. 2021; Giang et al. 2023), stakeholders reported that this could reduce trust if something happened which meant the caregiver may not be able to make that appointment:

…scheduling that [next care interaction], I mean there's been a couple times I've been late, and it unsettles him, but I apologise for being late and I think that breaks down a little bit of the trust sometimes’. (Home‐care worker, female, 8)

We therefore omitted this strategy from our theory.

#### 3.3.2 Sense of Control

**When a caregiver adopts a person‐led care approach and empowers the person (C), the person with dementia has a sense of being in control (M) and is more likely to accept assistance with personal care (O)**.

Having a sense of control was a mechanism for people with dementia to accept assistance, evidenced from 44 sources and 15 interviews. Acknowledging and addressing the person's need for control (Sloane et al. 2004) over the routine or situation (Kristensen and Peoples 2020; Roberto et al. 2024) was highlighted, along with the caregiver role in allowing or facilitating this (Volicer 2021). Power dynamics were highlighted by stakeholders, where empowering the person was viewed as a vital element to facilitate a sense of control, since care interactions usually reflected power imbalances with caregivers having more power:

So, I say right, you're in charge, you tell me what you're gonna do. Like, “oh, really?” …Oh, and I think that makes them a little bit more, more happy, I think. “Well, he's told me I'm in charge. And I thought you [caregiver] was in charge” (Home‐care worker, male, 10)

Caregiver strategies likely to create a sense of control included learning about the person and adapting to their preferences, offering choices (limited, with visual cues), establishing a joint goal, promoting independence/self‐care, seeking consent/assent, letting the person lead, carers telling the person what they are doing at each step to enable assent or refusal, and stopping when asked. Control could be overt such as the person with dementia giving instructions or consent (Giang et al. 2023; Henriques et al. 2019; Kobayashi et al. 2021), or more subtle, with the person knowing what was happening and going along with the care activity, or the caregiver working to the person's preferences (e.g., Davidson 2007; Faraday et al. 2021; Jung et al. 2024).

Some stakeholders, particularly family caregivers, thought that this mechanism was easier to enact with those with more cognitive ability and would become more difficult as dementia progressed, especially when giving choice to the person:

I make the decision …rather than giving a choice, you can't give people with Alzheimer's or dementia a choice, it has to be a pretty straight statement depending on the progression of the disease of course (Family caregiver, spouse, male 13)

However, others thought that a sense of control could be created whatever stage the person was at through strategies such as, adapting to the person's preferences and responding to the person and others:

Any way that we can help somebody to gain some of that control back is important. …there's lots of different ways of doing that. …telling people what's going to happen if they're not able to do it for themselves, supporting somebody to do as much as they can for themselves (Care home staff, female 4)

#### 3.3.3 Positive Connection

**When a caregiver knows the person's history and/or values the person as a human and approaches the person respectfully and genuinely and creates a warm, encouraging and friendly social environment (C), the person with dementia feels a sense of connection, belonging, inclusion and self‐worth (M) and is more likely to accept assistance with personal care (O)**.

Fifty‐five sources and 15 interviews provided evidence for the positive connection programme theory. Knowing the person meant the caregivers could go into the interaction armed with knowledge to utilise to make connections and provide care how the person may want it:

It would be helpful if there was a history of the person who actually was looking at because it does …make a difference. …Every person is different. That's what makes it so hard. …not everyone would want that, would like that so. But knowing the person that you're caring for, really does make a difference (Person with dementia, male 16)

A connection also came through as a prerequisite for care, in that care should not be thought of or mentioned until a social connection had been created (Thorsen and Nielsen 2023). Connection was also important throughout the care interaction (Faraday et al. 2021) to instil a sense of belonging (Cartwright et al. 2022). Caregiver strategies were numerous (Table 3) and included easily achievable actions such as knocking on the door, approaching the person from the front, smiling, making eye contact, greeting/announcing their arrival, being friendly and using the person's name. For example:

Every single room you go in, you tap on the door, you use that person's name, and you use your name. …you reintroduce yourself so on every occasion. You never enter anyone's room without knocking on the door first. …it's all about respect (Care home staff, female, 1)

However, family caregiver stakeholders found formal connection strategies such as greeting the person and using eye contact to be less relevant, since a strong connection was already in place.

More complex caregiver strategies for connection were also identified, such as the caregiver showing they are a safe person, being present in the moment, having fun together with the person, building rapport, being empathetic and validating the person by acknowledging and addressing issues. Two stakeholders mentioned connection working to build up a person's confidence, for example:

I sing and dance as well …you're in that situation where if you can make them laugh …If you can make ‘em (sic) happy …It's got to be some kind of build up because if you don't build confidence then what have you got? …You've got to be confident; they've got to be confident (Home care worker, male, 10)

#### 3.3.4 Care Feels Manageable

**When the caregiver responds to the person in the moment and/or simplifies the sound of, or processes of, the care activity (C), the person with dementia feels more capable of managing the care activity (M) and is more likely to accept assistance with personal care (O)**.

Forty‐three sources and 14 interviews evidenced the care feels manageable programme theory. Caregiver strategies (Table 3) included being flexible and adapting to the person during each interaction, guiding the person through the activity in small steps, building the person's confidence, employing person‐led care, minimising the sound of care, following the person's usual routine, using known terminology, offering a different mode of care, making sure the person is in an appropriate position and offering limited information or choices.

Appropriate speed of care was a key strategy to make care feel manageable (e.g., Gilmore‐Bykovskyi 2015; James et al. 2020; Rey et al. 2020; Faraday et al. 2021):

it has to be the patient's pace. …You can't, and you shouldn't expect them to go at the caregiver's pace …you're just setting everything up for disaster. …otherwise, …there is that lack of respect, that lack of dignity there and he's no longer being treated as another human being (Family caregiver, daughter, female, 15)

There were mixed views on whether appropriate pace should ever be quicker for those who preferred care to be quick (Jung et al. 2024) or who could be distressed by care or cold, with some stakeholders aware that speed may be best option in some circumstances:

…unless you need to quickly do it because they're going to wander off. Then you need to be quick. …It's about read the circumstances. …if you've got someone who wants to go slow and they're happy for personal care, then you can go slow. You can give time, but someone who's going to let you do it once and that's it. That's your opportunity. Quickly get that pad in. …it depends on the person (Home care worker, female, 9)

One stakeholder thought that there would be no need to ever speed up care assistance, if other relational, trust and safety aspects were in place:

Very rarely have I found that (going faster) to be the most appropriate way forward, although …I can understand that we minimise the stress you know and so we do it fast. But actually, if we often do all of those other things first, we don't have to do it fast (Professional, female, 14)

A few stakeholders thought that care feeling manageable would depend on the cognitive abilities of the person, with more cognitive ability making care more manageable.

#### 3.3.5 Working Together

**When a caregiver views the person as a partner in the care interaction and works together with them (C), the person with dementia feels part of a collaborative activity (a shared enterprise) generating a sense of achievement (M) and is more likely to accept assistance with personal care (O)**.

This programme theory was evidenced by 20 sources and 14 interviews. Working together was reported to enable the person to gain a sense of achievement and feel part of something (Cartwright et al. 2022). This programme theory emulates a partnership sentiment, doing with, rather than to (Ostaszkiewicz, Dickson‐Swift, et al. 2020; Backhouse, Jeon, et al. 2024; Westerberg and Strandberg 2007). A home care worker uses a maths quiz analogy to sum up the working together theory:

I hate maths, so if someone came to me and said ‘right, do you want to do this math quiz?’ I'm like, ‘no’. But then if they say, shall we go and sit down and do this math quiz? I'll be like, yeah all right then, because there's both of you. You're going to have that assistance, help with it. Not only that, it could be a fun, enjoyable experience. …it's not going to be too much on my shoulders (Home care worker, female 8)

Caregiver strategies include starting an activity and handing it over to the person to complete, establishing a shared goal, telling the person what they are doing, giving the person a care item to hold, using the guiding hand technique, involving the person and working with them and using inclusive and inviting terms such as ‘shall we?’ and ‘can you help me?’

Asking the person for help (James et al. 2020; Jablonski‐Jaudon et al. 2016; Snow 2022) was viewed as particularly useful by several stakeholders to enable the person a sense of self‐worth and accomplishment

…could you help me do this? I think is a really good thing because it gives a person a sense of purpose and like that an achievement and they've done something …it's not nice never being able to help other people or to be able to do something (Family caregiver, daughter, female, 15)

Some stakeholders thought working together was more feasible when the person was less cognitively impaired.

#### 3.3.6 Comfort

**When the caregiver acts in a way that prioritises the person's experience of care (C), the person with dementia feels increased comfort (physical, sensory, emotional) (M) and is more likely to accept assistance with personal care (O)**.

The comfort programme theory was evidenced by 49 sources and 15 interviews. Comfort included physical, emotional and sensory comfort (Volicer 2021; Davidson 2007; Buse and Twigg 2018). Caregiver strategies for emotional comfort included connecting with the person, reassuring the person, positioning themselves innocuously, praising the person, making care enjoyable, adapting to the person's preferences, not using elderspeak, going along with the person's reality and validating the person:

you [should] go along with their story. Like if they're saying their dad's home at teatime, their dad's home at teatime, that's what's happening. Because a lot of people correct them, and then they get more distressed and they can't get their head around it (Home care worker, female, 9)

Strategies for sensory comfort included the caregiver using therapeutic touch, employing music/singing, using touch, holding hands or stroking hair as appropriate to provide pleasurable care and adapting the environment (lights, noise, busyness) to the person's needs and/or preferences:

We've been in to do care in a resident's room, and we sat either side of them on the bed, just gently touching the hand so they know [you're] with them and talking quietly to them. …just reassure them, that reassurance comes and goes a long way doesn't it? And then we can gradually …explain to him what we're going to do’ (Professional, female 6)

Care strategies for physical comfort included addressing pain before care, being gentle (e.g., with touch, water spray and toothbrush) and ensuring appropriate temperature/s (of the room, water and/or person):

…sometimes people may be in pain, so have they had any pain relief prior to personal care which can make a big difference. …we've been to a (care) home …where you know they filled bowl with water. But the water was very tepid. …And then putting it on their skin, they've been warm in bed and then a cold flannel. …you're not really going to get a good result, are you?’ (Professional, female 5)

Some stakeholders thought that some physical comfort strategies such as checking the water temperature were basic and part of usual care.

#### 3.3.7 Needs Are Known and Addressed

**When the caregiver assesses the person for cues and clues of unmet needs and acts to address them before introducing or starting care (C), the person with dementia has a sense of peace and reassurance where their needs are known and met (M) and is more likely to accept assistance with personal care (O)**.

This programme theory was evidenced by 21 sources and 15 interviews. The importance of this theory as a prerequisite for all care activities was explained by a professional:

You can't think if you're distracted by something that's basic. …if you're hungry, you're not concentrating. If you're thirsty, you're aware of being thirsty. If you're in pain, that takes away all of your ability to think. (Professional, female, 14)

Caregiver strategies included finding out about the person, making sure sensory supports are in place (hearing aid, glasses, teeth), addressing any physical discomfort before introducing or starting care (including hunger or thirst), resolving emotional distress before introducing or starting care, ensuring privacy and dignity, and considering and trying to address symptoms (pain, depression, delusions).

However, the need to go beyond just meeting a person's needs, prioritising the relationship and being there for the person (Kobayashi et al. 2021; Thorsen and Nielsen 2023) was highlighted:

You can meet someone's needs, but you can only really care for someone you know, because meeting someone's needs is very different. …A different approach really cause you know my needs are I need to be fed. I need to have some intervention with personal care. I need a pad change. But caring about me as you change my pad is different. …it's the jewel in the crown, the art of care. …It's what takes it from a task to a joint adventure in getting something done (Care home staff, female, 3)

This demonstrates the importance of the interrelated nature of the eight programme theories in creating holistic personal care assistance (see Figure 2).

#### 3.3.8 Engaging With the Care Activity (Or Something Non‐Care Related)

**When the caregiver helps to focus the person's attention on the care activity (or on something non‐care related) (C), the person with dementia feels engaged with what is happening during the care activity (or with something else if care can be distressing or unsettling) (M) and is more likely to accept assistance with personal care (O)**.

The engaging with the care activity (or something non‐care related) programme theory was evidenced by 48 sources and 14 interviews. To promote engagement with the care activity, caregiver strategies included explaining what is going to happen next and waiting for the person to digest this, using clear, short instructions, taking time and repeating themselves if needed, simplifying care, sitting the person up (if able), removing distractions, promoting self‐care, validating and acknowledging the person's experience, using non‐verbal communication and promoting contact with care objects. A family caregiver talks through some of their strategies:

their [the person with dementia's] attention needs to be solely on that activity at that time, Like cleaning her teeth, I need to make sure that [Name] actually cleans her teeth and doesn't get distracted by wiping out the basin with the toothbrush, which she does …a demonstration to clean the teeth …keep my sentences short and directly to the point …I have to repeat it several times (Family caregiver, spouse, male 13)

To motivate the person, caregiver strategies included encouraging, prompting, praising, entering the person's reality and offering or pointing out a reward after care:

Every step of the way, we're …encouraging them and where they need a little prompt. We will give them the prompt, but obviously all the caregivers know they do still have a right to refuse something. …cup of tea and a biscuit and you can get most things done (Professional, female, 7)

To promote the person's engagement with something non‐care related caregiver strategies included giving the person something to hold (e.g., a comfort item), using music/singing and talking about something else during care:

the music takes them to a different place so that what is physically happening doesn't distress them (Care home staff, female, 3)

Most data and stakeholders referred to ‘distraction’ (e.g., Moniz‐Cook et al. 2003; Jablonski, Therrien, Mahoney, et al. 2011; Chou et al. 2016) when getting the person's attention away from the care activity, however one stakeholder and two sources (Buse and Twigg 2018; Jung et al. 2024) used the term engagement. The stakeholder explained that distraction was really engagement:

…people will only get dressed if you're singing and they'll respond …because they're tuning into you that you're then tuning into each other. So, it's not actually always a distraction. It's sometimes a technique for engagement. And so is holding an item (Professional, female, 14)

Public involvement representatives also endorsed the term engagement over distraction, so the team adopted the term. Stakeholders thought that engagement in the care activity could depend on the severity of a person's dementia, with those more advanced less able to engage.

#### 3.3.9 Obligation

A sense of obligation was also a person with dementia mechanism response that was generated from data extracted from our sources (Roberto et al. 2024; Villar et al. 2022; Backhouse, Jeon, et al. 2022; Backhouse and Ruston 2022; Villar et al. 2022; Apesoa‐Varano 2020; James et al. 2020; O'Brien et al. 2020; Jablonski et al. 2018; Buse and Twigg 2018; Jablonski‐Jaudon et al. 2016; Gilmore‐Bykovskyi 2015; Kutsumi et al. 2009; Chang and Roberts 2008; Nagahama et al. 2022; Graneheim et al. 2005; Westerberg and Strandberg 2007).

IF the caregiver persuades, coaxes or persists, uses firm entitled commands, an authority figure, negative reinforcement or restraint (continuing with care after a refusal, forced care, sedation, hand holding) THEN the person with dementia will be more likely to accept care assistance BECAUSE they feel obligated or compelled to do so. Team meetings and stakeholder interviews showed this programme theory to be undesirable. Some stakeholders thought it was not acceptable, and others thought it may be useful as a last resort in some scenarios. For example, feeling obligated due to persuasion or firm commands could be the least restrictive options compared to medication use or controlled restraint. Stakeholders suggested that family caregivers may sometimes employ obligation‐inducing strategies such as persuading, but this would contravene professional codes of conduct. Some stakeholders stated that these approaches would make the person with dementia less likely to accept care assistance. Additionally, such pressure might work once but erode trust for subsequent occasions. We have left this theory out of our model so as not to highlight it as a desirable mechanism to elicit.

## 4 Discussion

### 4.1 Summary of Main Findings

This realist synthesis has presented eight interrelated programme theories, in CMO configurations, identifying strategies and mechanisms of interventions between caregivers and people with dementia that contribute to reducing refusals of care/increasing acceptance of personal care assistance. The overarching theory, with all other theories building into it, showed trust in the caregiver and a feeling of safety to be key mechanism responses of people with dementia leading to potential care acceptance and therefore constitutes the ultimate goal of personal care assistance. Caregivers need to use multiple strategies to provide holistic personal care assistance to people with dementia. If caregivers can ensure care feels manageable, the person with dementia has a sense of control, and there is a positive connection throughout care interactions, then trust and a feeling of safety are more likely. Working together with the person, making sure they are comfortable, their needs are known and addressed and they are engaging with the care activity (or something non‐care related) will support the aforementioned mechanism responses. Our findings provide a toolkit of caregiver strategies to make personal care assistance more likely to be accepted by people with dementia.

Sources contained limited information about ‘for whom’ the theories would work beyond caregiver actions and mechanism responses from people with dementia; however, stakeholders thought four theories (sense of control, care feels manageable, working together, engaging with the care activity (or something non‐care related)) would be more applicable for people with dementia with more cognitive ability. Formal strategies of connection were viewed as less relevant for family caregivers/spouses.

Our findings are of relevance across settings, including for nursing practice and education. Personal care assistance has key relevance to nurses, particularly care home and student nurses (Santo et al. 2019), but also to those working in other settings who may provide less hands‐on personal care but support others providing direct care. When people with dementia refuse assistance, nurses are often consulted for advice; in this way, even those in team leadership roles have a key position in supporting personal care assistance. Additionally, assisting with personal care offers time for nurses to provide holistic care, build bonds and pick up on important cues and changes in the care recipient's condition (Aston 2013).

### 4.2 Self‐Determination Theory

Once IPTs were developed, Self‐determination Theory (Ryan and Deci 2017, 2020) was identified as a compatible midrange theory to support CMO development and offer broader explanations for our programme theories. Self‐determination theory is a psychological theory. It has been applied in nursing in relation to nurse education (e.g., Hosseini et al. (2022) via examination of motivation of nursing students), nursing practice development (Dufour and Duhoux 2024) and intervention development (Huang et al. 2022). Self Determination Theory sets out three innate psychological needs of

1. Competence—feeling able to operate effectively. If challenges are too difficult or there is negative feedback, competence wanes.
2. Autonomy—feeling that you can act to your preferences, a sense of voluntariness, behaviours are self‐endorsed and congruent with one's authentic values.
3. Relatedness—feeling socially connected, cared for, belonging and significant among others.

Our theories: care feels manageable, sense of control and positive connection closely echo these psychological needs. Self Determination Theory suggests that when these needs are supported, they produce improved self‐motivation and wellbeing. When these are hindered, they lead to reduced motivation and wellbeing. In dementia, a person's abilities to support their own competence, autonomy and relatedness are often diminished, making the caregiver role vital to support these factors. In this way, our theories have a role in improving self‐motivation and wellbeing via caregiver actions. Conceivably, we can motivate people with dementia to accept care assistance through maximising their real or perceived control in the interaction (autonomy), making sure they feel the care activity is manageable for them (competence) and connecting with them in a positive way (relatedness). These innate psychological needs are usually determined by a person themselves; however, this can be hard for a person with dementia. Instead, they must rely on their caregiver/s to help meet these innate psychological needs; therefore, trust and safety become paramount. Self‐determination theory extended our thinking regarding the programme theories in relation to motivation and why trust and safety are so important for people with dementia in particular.

### 4.3 Strengths, Limitations and Future Research Directions

Strengths of this research include the number and variety of sources drawn on, stakeholder involvement through interviews and public involvement, alignment with a mid‐range theory and the potential practical nature of the findings to inform practice. However, including so many sources can lead to a wide range of contexts, populations, rigour levels and document types (grey literature, qualitative, quantitative), making theoretical coherence difficult. Other limitations include the decisions to focus closely on personal care interactions and not look beyond mechanism responses of people with dementia, therefore missing caregiver mechanism responses within this synthesis and wider contextual factors. For example, it is likely that settings such as care homes would have limited time to implement some identified strategies, so some practices may be good in theory and difficult in practice. Additionally, all interviewees were White British, and only one had dementia. Multiple sources were assessed as having low rigour, which may affect the strength of our findings. Future directions include putting the programme theories into practice combined with real‐life evaluation to gain evidence regarding their usefulness in clinical practice. We will be using the learning from this synthesis to feed into the development of training for paid and unpaid caregivers of people with dementia who require assistance with personal care activities.

### 4.4 Comparison With Existing Literature

Evidence from systematic reviews shows that playing recorded music, communication strategies and different bathing techniques, including person‐centred bathing, can reduce refusals of care (Konno et al. 2014, 2024; Backhouse et al. 2020). This synthesis endorses these strategies and adds valuable information about *how* personal care assistance may be improved by caregivers and *why* people with dementia may accept assistance.

Broad contextual factors were beyond the scope of our study, but previous realist reviews provide important insights into aspects which could impact on the feasibility of our theories in practice. For example, in care homes, there may be a need for ongoing staff teaching and feedback, personalised care planning reflecting the cognitive and physical abilities of residents, and integration of intervention actions into everyday practices (Goodman et al. 2017). In hospitals, staff viewing behaviours that challenge as expressions of unmet needs was a context for addressing those needs (Handley et al. 2017), this relates to our ‘needs are known and addressed’ theory and demonstrates the wider role of caregiver perspectives for influencing and activating person with dementia mechanisms.

van der Weide et al. (2023) conducted a realist review examining interventions for supporting autonomy for people with dementia in nursing homes and included limiting choices or creating visual choices, so residents feel confident and less overwhelmed, and residents needing to feel acknowledged and build trust with staff to feel like they are relaxed and belong. Although the review did not focus on personal care assistance, these themes resonate with and endorse the sense of control and trust and safety theories.

As with other research in this area (Prizer and Zimmerman 2018; Cartwright et al. 2022), our findings align strongly with Kitwood's concept of person‐centred care (1997, 1998). Person‐led care (a term used by some stakeholders) came through strongly, as seen in the notion that meeting the person with dementia's needs is not enough; caregivers also need to treat the person as a human being, connecting with and valuing them. This was endorsed by several included sources (e.g., Kobayashi et al. 2021; Thorsen and Nielsen 2023) and interviews. These findings echo existing literature showing that family caregivers regarded home‐care workers meeting a person with dementia's needs as lower priority than the companionship and emotional and social support they may also offer (Pollock et al. 2021).

Our findings support using the collective ‘we’ such as ‘shall we?’ to reflect partnership working, praise to comfort and motivate, and using short terms and simple vocabulary to engage the person. However, these can be viewed as contentious in dementia care literature as part of elderspeak, which has been found to increase refusals of care (Zhang et al. 2020). However, some aspects of elderspeak can improve comprehension such as short words or simple sentences (Shaw and Gordon 2021). Our synthesis shows that these and the aforementioned aspects can work positively in personal care interactions, yet caution should be exercised to make sure their use is not patronising.

## 5 Conclusion and Recommendations

This is, to our knowledge, the first realist synthesis examining how personal care interactions between caregivers and people with dementia can be improved to reduce refusals of care. Eight interrelated programme theories were developed, which suggest that building *trust and safety* in personal care interactions should be the main goal of providing personal care assistance for people with dementia. Person with dementia mechanism responses of trust and safety can be built directly, but also through having *a sense of control, care feeling manageable and positive connection*; these can be further bolstered by *working together, feeling comfort, needs being known and addressed and engagement with the care activity* or something non‐care related. The findings provide theoretical ideas linked to practical care strategies which could be helpful for those directly assisting people with dementia or working to improve personal care practices for people with dementia, such as nurses and paid and unpaid caregivers, and could increase positive outcomes. More research is needed to test the programme theories in real‐life situations. Ultimately, meeting a person with dementia's physical personal care needs is not enough; clinically, those providing assistance need to also treat the person as a valued human being. Relationships are fundamental to personal care assistance. *A joint adventure is required*, *with trust and safety the central goal*.

## Ethics Statement

This research received a favourable ethical opinion from the Social Care Research Ethics Committee IRAS project ID: 338274, REC reference: 24/IEC08/0007; Date 08.04.2024.

## Conflicts of Interest

The authors declare no conflicts of interest.

## Supporting information


**Data S1:** jan70204‐sup‐0001‐DataS1.docx.

**Data S2:** jan70204‐sup‐0002‐DataS2.docx.

## Data Availability

The data that support the findings of this study are available from the corresponding author upon reasonable request.
