# Supplementary material for: How Can We Improve Personal Care Interactions to Reduce Care Refusals From People With Dementia? A Realist Synthesis
Source: J Adv Nurs. 2025 Sep 18;82(5):4895–926. doi: 10.1111/jan.70204 (PMC13069214; doi:10.1111/jan.70204)
Supplement: Supplementary file 2 — Data S2: jan70204‐sup‐0002‐DataS2.docx. [file JAN-82-4895-s001.docx]

**Supplementary Material**

**Full search syntax: 22.04.2024 in OVID MEDLINE, OVID EMBASE, EBSCO PsycINFO, EBSCO CINAHL Ultimate, Cochrane CENTRAL register of controlled Trials, and Web of Science.**

(("Dementia" or "Alzheimer*" or "Cognitive decline" or "Cognitive impair*" or "Lewy bod*") and ("personal care" or "ADLs" or "activities of daily living" or "bath*" or "shower*" or "wash*" or "toilet*" or "dress*" or "morning routine" or "shav*" or "eat*" or "medication*" or "drink*" or "nail*" or "hair" or "care" or "teeth" or "oral hygiene" or "skin care" or "feed*") and ("resistan*" or "refus*" or "reject*" or "non-complian*" or "noncomplian*" or "complian*" or "uncooperative" or "obstreperous" or "accept*"))

**Formal search: trust and safety 20.12.2024 OVID MEDLINE and EMBASE**

(("Dementia" or "Alzheimer*" or "Cognitive decline" or "Cognitive impair*" or "Lewy bod*") and ("personal care" or "ADLs" or "activities of daily living" or "bath*" or "shower*" or "wash*" or "toilet*" or "dress*" or "morning routine" or "shav*" or "eat*" or "medication*" or "drink*" or "nail*" or "hair" or "care" or "teeth" or "oral hygiene" or "skin care" or "feed*") and ("trust” or “safety”))

98 results - OVID MEDLINE 40 and EMBASE 58

**Table A: Strategies, references, and connected theory**

| **Reference** | **Intervention strategy** | **Trust and safety** | **Sense of control** | **Positive connection** | **Care feels manageable** | **Working together**                **X**                                    **X**                                        **X**    **X**                                                          **X**            **X**  **X**                          **X**  **X**        **X**  **X**    **X**        **X**  **X**                                              **X**                    **X**                              **X**              **X**                  **X**                                                                        **X**                **X**        **X**  **X**        **X**            **X**                                **X**                                          **X**    **20** | **Comfort** | **Needs are known and addressed** | **Engaging with care (or something else)** |
| --- | --- | --- | --- | --- | --- | --- | --- | --- | --- |
| Amella, 2002 | If carers are inflexible and bothered rather than flexible and cool |  |  |  | X |  |  |  |  |
| Apesoa-Varano, 2020 | If the carer waits and tries again |  | X |  |  |  | X |  |  |
|  | If the carer makes the care activity into a pamper session |  |  | X |  |  |  |  |  |
|  | If the carer taps into the person's past interests |  |  | X |  |  |  |  |  |
|  | If the carer demonstrates/mimes the activity |  |  |  |  |  |  |  | X |
|  | If the carer explains why the care activity is needed |  |  |  |  |  |  |  | X |
| Ashida et al., 2024 | If the carer tries a different time |  | X |  |  |  | X |  |  |
|  | If the carer makes the activity part of a routine |  |  | X |  |  |  |  |  |
| Astorga et al., 2023 | If the carer verbally prompts the person |  |  |  |  |  |  |  | X |
|  | If the carer plays some music |  |  |  |  |  | X |  |  |
| Backhouse et al., 2020 | If the carer plays music during care |  |  | X |  |  | X |  | X |
|  | If the carer offers different bathing options | X |  | X |  |  |  |  |  |
|  | If the carer reduces elderspeak |  |  |  |  |  | X |  |  |
|  | If the carer reduces negative communications |  |  |  |  |  | X |  |  |
| Backhouse et al., 2024^b^ | If the carer invites the person to be part of the interaction |  | X |  |  |  |  |  |  |
|  | If the carer seeks permission for care |  | X |  |  |  |  |  |  |
|  | If the carer emanates a sense of togetherness |  |  |  |  | X |  |  |  |
|  | If the carer puts the person at their ease |  |  | X |  |  |  |  |  |
|  | If the carer praises the person |  |  |  |  |  | X |  | X |
|  | If the carer reassures the person | X |  | X |  |  | X |  |  |
|  | If the carer demonstrates the activity to the person |  |  |  |  |  |  |  | X |
|  | If the carer guides the person through the care activity |  |  |  | X |  |  |  |  |
|  | If the carer uses the instruct-endorse communication technique |  |  |  | X |  | X |  |  |
|  | If the carer encourages the person |  |  |  |  |  |  |  | X |
|  | If the carer Prompts the person |  |  |  |  |  |  |  | X |
|  | If the carer uses non-verbal communication |  |  |  |  |  |  |  | X |
|  | If the carer describes care in a minimising way |  |  |  | X |  |  |  |  |
|  | If the carer uses clear instructions |  |  |  |  |  |  |  | X |
|  | If the carer does not hurry the person |  |  |  | X |  |  |  |  |
|  | If the carer positions themselves innocuously | X |  |  |  |  | X |  |  |
| Backhouse et al., 2022^a^ | If carers leave and return later |  | X |  | X |  |  |  |  |
|  | If carers change the timing of care |  |  |  | X |  |  |  |  |
|  | If carer address unmet needs before care |  |  |  |  |  |  | X |  |
|  | If carers encourage the person |  |  |  |  |  |  |  | X |
|  | If carers explain what they are doing and why |  |  |  |  |  |  |  | X |
|  | If carers describe the care activity in a minimising way |  |  |  | X |  |  |  |  |
|  | If carers provide reduced care |  |  |  | X |  |  |  |  |
|  | If carers change the mode of care |  |  |  | X |  |  |  |  |
|  | If carers use a routine |  |  |  | X |  |  |  |  |
|  | If carers distract the person |  |  |  |  |  |  |  | X |
|  | If carers create time for care |  |  |  | X |  |  |  |  |
|  | If carers get another carer involved - two carers |  |  |  | X |  |  |  |  |
|  | If carers leave some care uncompleted |  |  |  | X |  |  |  |  |
|  | If carers have a cheerful approach |  |  | X |  |  |  |  |  |
| Backhouse and Ruston, 2022 | If the carer gets another carer to help |  |  |  | X |  |  |  |  |
|  | If the carer tries at different times of day |  |  |  | X |  |  |  |  |
|  | If the carer distracts the person (cup of tea, draw on person's interests) |  |  |  |  |  |  |  | X |
|  | If the carer involves the person in the care activity |  |  |  |  | X |  |  |  |
|  | If the carer starts with a different aspect of the person's required care |  |  |  | X |  |  |  |  |
|  | If the person leaves and tries again later |  | X |  | X |  |  |  |  |
|  | If the carer uses the person's routine |  |  |  | X |  |  |  |  |
|  | If the carer has a calm mannerism |  |  | X |  |  |  |  |  |
|  | If the carer positions themselves at the person's level | X |  | X |  |  |  |  |  |
|  | If the carer uses humour |  |  | X |  |  |  |  |  |
|  | If the carer explains what they are doing |  |  |  |  |  |  |  | X |
|  | if the carer praises the person |  |  |  |  |  | X |  | X |
|  | If the carer minimises the sound of the task/talks the task up |  |  |  | X |  |  |  |  |
|  | If the carer offers choices rather than yes/no questions |  | X |  |  |  |  |  |  |
|  | If the carer offers limited choices |  | X |  |  |  |  |  |  |
|  | If the carer adapts their tone of voice |  |  | X |  |  |  |  |  |
| Boersma et al., 2017 | If the carer greets the person one-on-one - thinking about intonation and timing |  |  | X |  |  |  |  |  |
|  | If the carer appeals to long-term memory through singing or a conversation about objects from the past |  |  | X |  |  |  |  |  |
|  | If the carer Communicates through music/humming by singing together, reciting poems and rhymes |  |  | X |  |  | X |  | X |
|  | If the carer says goodbye | X |  |  |  |  |  |  |  |
| Bray et al., 2021 | If background music is played during care |  |  | X |  |  | X |  | X |
|  | If carers provide loving touch |  |  | X |  |  | X |  |  |
| Buse and Twigg 2018 | If the carer continuously reassures person | X |  | X |  |  | X |  |  |
|  | If the carer has a clam approach |  |  | X |  |  |  |  |  |
|  | If appropriate, loose, stretchy and easy clothing is used |  |  |  | X |  |  |  |  |
|  | If the carer takes time when assisting with dressing |  |  |  | X |  |  |  | X |
|  | If the carer explains the care activity |  |  |  |  |  |  |  | X |
|  | If the carer leaves the person for a little while |  | X |  | X |  |  |  |  |
|  | Try a different carer |  |  |  | X |  |  |  |  |
|  | If the carer uses emotion to manipulate the person |  |  |  |  |  |  |  |  |
|  | If the carer uses the care interaction for a chat with the person |  |  | X |  |  |  |  |  |
|  | If the carer complements the person |  |  |  |  |  | X |  | X |
|  | If the carer engages the person in the activity on a sensory level - feeling the material of clothes |  |  |  |  |  |  |  | X |
|  | If the carer offers person choices |  | X |  |  |  |  |  |  |
|  | If the carer maintains the person's usual habits/identity |  |  |  | X |  |  |  |  |
| Cartwright et al., 2022 | If the carer promotes choice - visual and verbal |  | X |  |  |  |  |  |  |
|  | If the carer allows time for the person to choose |  | X |  |  |  |  |  | X |
|  | If the carer engages with the person about the care and socially inc. body language/listening/validation/ belonging/ fun - NOT invalidating/ignoring/mocking/withholding reassurance |  |  | X |  |  |  |  |  |
|  | If the carer prompts/encourages verbally and physically - empowerment /facilitation |  |  |  | X |  |  |  | X |
|  | If the carer celebrates the person achievement |  |  | X |  | X |  |  |  |
| Chang and Roberts 2008 | If the carer tries again later |  | X |  | X |  |  |  |  |
|  | If the carer verbally encourages the person |  |  |  |  |  |  |  | X |
|  | If the carer offers different food/choices |  |  |  | X |  |  |  |  |
|  | If care is undertaken in an appropriate space and position |  |  |  |  |  |  |  | X |
| Chou et al., 2016 | If a carer distracts the person with a positive image (individualized) |  |  | X |  |  |  |  | X |
| Davidson, 2007 | If the carer addresses discomfort physical or emotional |  |  |  |  |  | X | X |  |
|  | If the carer adapts tasks to meet person’s ability |  |  |  | X |  |  |  |  |
|  | If the carer involves person |  |  |  |  | X |  |  |  |
|  | If the carer allows time for participation |  |  |  | X |  |  |  |  |
|  | If the carer encourages person |  |  |  |  |  |  |  | X |
|  | If the carer communicates simple steps |  |  |  | X |  |  |  |  |
|  | If the carer removes/reduces choices |  | X |  |  |  |  |  |  |
|  | If the carer knows person's preferences |  | X |  |  |  |  |  |  |
|  | If the carer reduces distractions |  |  |  |  |  |  |  | X |
|  | If a familiar carer assists the person |  |  | X |  |  |  |  |  |
|  | If the carer uses a familiar routine |  |  |  | X |  |  |  |  |
| Del and Palace, 2016 | If the carer shows the person a video of their loved one with a greeting and message of support, comfort, love and affection |  |  | X |  |  | X |  |  |
| The Eden Alternative, UK, 2025 | If the carer promotes independence |  | X |  |  |  |  |  |  |
|  | If the carer changes the time of care |  |  |  | X |  |  |  |  |
|  | If the carer offers a cup of tea first before care |  |  |  |  |  | X |  |  |
|  | If the carer slows down their approach |  |  |  | X |  |  |  |  |
| Faraday et al., 2021 | If the carer interacts, builds relationships and social connections during mealtimes |  |  | X |  |  |  |  |  |
|  | If the carer allows adequate time to feed |  |  |  | X |  |  |  |  |
|  | If the right amount of care is provided for the person's ability |  |  |  | X |  |  |  |  |
|  | If personal preferences / choices are catered for |  | X |  |  |  |  |  |  |
|  | If the carer promotes independence - encouraging, prompting, reinforcement |  |  |  |  |  |  |  | X |
|  | If the carer provides care at the optimal time of day for the person |  |  |  | X |  |  |  |  |
|  | If the carer provides visual, tactile (napkin to mouth), and verbal cues |  |  |  |  |  |  |  | X |
|  | If the carer removes distractions |  |  |  |  |  |  |  | X |
| Galindo-Garre, 2015 | If the carer addresses the person's depression |  |  |  |  |  |  | X |  |
|  | If the carer helps the person understand what is going to happen/is happening | X | X |  |  |  |  |  | X |
| Gaugler et al., 2016 | If the carer connects with the person |  |  | X |  |  |  |  |  |
|  | If the carer approaches from the front |  |  | X |  |  |  |  |  |
|  | If the carer stands to the side of the person | X |  | X |  |  |  |  |  |
|  | If the carer offers the person their hand |  |  | X |  |  |  |  |  |
|  | If the carer slows down their approach |  |  |  | X |  |  |  |  |
|  | If the carer plays some soft, soothing music |  |  | X |  |  | X |  |  |
|  | If the carer leaves the person and returns later |  | X |  | X |  |  |  |  |
| Giang et al., 2023 | If the carer announces their presence |  |  | X |  |  |  |  |  |
|  | If carers announce and describe the care procedures to the person with dementia during all care processes | X | X |  |  |  |  |  | X |
|  | If the carer uses professional and progressive touch |  |  | X |  |  | X |  | X |
|  | If the carer makes sure the person adopts an upright position - if possible |  |  |  |  |  |  |  | X |
|  | If the carer looks at the person face-to-face, horizontally, sustained, and in close proximity |  |  | X |  |  |  |  |  |
|  | If the carer establishes a relationship with the person |  |  | X |  |  |  |  |  |
|  | If the carer seeks consent for the care |  | X |  |  |  |  |  |  |
|  | If the carer bids farewell after the care | X |  |  |  |  |  |  |  |
|  | If the carer commits to a future care encounter | X |  |  |  |  |  |  |  |
| Gilmore-Bykovskyi, 2015 | If the carer outpaces the person |  |  |  | X |  |  |  |  |
|  | If the carer gives lots of positive face gestures and cues |  |  | X |  |  |  |  |  |
|  | If the carer gives the person space to be independent |  |  |  | X |  |  |  |  |
|  | If the carer gives too much or too little assistance |  |  |  | X |  |  |  |  |
|  | If the carer gives person-centred interaction |  | X |  |  |  |  |  |  |
| Gjellestad et al., 2022 | If the carer builds up familiarity with the person over time |  |  | X |  |  |  |  |  |
|  | If the carer has time to spend with the person |  |  |  | X |  |  |  |  |
|  | If the carer uses time and make them feel safe, hold their hands, and let them say stop when they feel it is too much | X |  |  |  |  |  |  |  |
|  | If the carer goes in and sits and talks with the person first |  |  | X |  |  |  |  |  |
| Gjellestad et al., 2023 | If the carer respects the person's preferences/choices |  | X |  |  |  |  |  |  |
|  | If the carer makes the person feel safe | X |  |  |  |  |  |  |  |
|  | If familiar carers assist the person |  |  | X |  |  |  |  |  |
|  | If all carers conduct tasks the same way |  |  |  | X |  |  |  |  |
|  | If the carer spends time before the care interaction (serve coffee/cake) |  |  | X |  |  |  |  |  |
|  | If the carer creates a good atmosphere |  |  | X |  |  |  |  |  |
|  | If the carer has a calm approach |  |  | X |  |  |  |  |  |
|  | If the carer uses hand-leading |  |  |  |  | X |  |  |  |
|  | If the carer uses eye contact |  |  | X |  |  |  |  |  |
|  | If two carers assist the person and one gives emotional support and one doing the practical work |  |  |  | X |  |  |  |  |
|  | If carers use clear communication - one or a few words to explain what was going to happen |  |  |  |  |  |  |  | X |
|  | If one carer is refused, another carer tries |  |  |  | X |  |  |  |  |
|  | If the same carers attend the person |  |  | X |  |  |  |  |  |
|  | If a carer goes back and tries again later |  | X |  | X |  |  |  |  |
|  | If family members encourage the person or be there during care |  |  | X |  |  |  |  |  |
|  | If carers adjust to the person routine |  |  |  | X |  |  |  |  |
| Graneheim et al., 2005 | If the carer appears capable |  |  |  | X |  |  |  |  |
| Gutman, Karbakhsh, et al., 2021 | If the carer introduces a screen of garden motion pictures |  |  |  |  |  |  |  | X |
| Gutman Vashisht, et al., 2021 | If the carer introduces a screen of garden motion pictures and prompts the person to look at it |  |  |  |  |  |  |  | X |
| Hammer et al., 2011^a^ | If the carer invites the person to join the communication through singing |  |  | X |  |  |  |  |  |
|  | If the carer uses eye contact |  |  | X |  |  |  |  |  |
|  | If the carer uses non-verbal communication (holds up a shirt) |  |  |  | X |  |  |  |  |
|  | If the carer invites the person to join the communication through singing |  |  | X |  |  |  |  |  |
|  | If the carer avoids eye contact if the person is resisting |  |  | X |  |  |  |  |  |
|  | If the carer is present and engaging with the person |  |  | X |  |  |  |  |  |
| Hammer et al., 2011^b^ | If the carer uses music therapeutic caregiving (MTC) - singing during care |  |  | X |  |  | X |  |  |
| Hanson et al., 2023 | If the carer has awareness of the whole person and their likes/dislikes |  | X |  |  |  |  |  |  |
|  | If the carer makes use of sensory supports with music, aromas, activities, and calming environment |  |  |  |  |  | X |  |  |
|  | if the carer responds to promote comfort when people with dementia express distress |  |  | X |  |  | X |  |  |
|  | If the carer tries to work out what the person may be feeling or communicating |  |  |  |  |  |  | X |  |
|  | If the carer addresses any pain the person may be in (pain assessment and pain rating scales for dementia, non-medication interventions, and safe use of pain medication) |  |  |  |  |  | X | X |  |
| Henriques et al., 2019 | If the carer announces their arrival |  |  | X |  |  |  |  |  |
|  | If the carer uses the person's name |  |  | X |  |  |  |  |  |
|  | If the carer uses their gaze, speech, and touch to build a relationship |  |  | X |  |  |  |  |  |
|  | If the carer seeks consent |  | X |  |  |  |  |  |  |
|  | If the carer leaves a positive impression of the relationship and of the care in the emotional memory of the person |  |  | X |  |  | X |  |  |
|  | If carers say farewell to person and schedule when they would meet next | X |  |  |  |  |  |  |  |
|  | If the carer tells the person the reason for the encounter |  |  |  |  |  |  |  | X |
|  | If the carer uses a calm tone and approach |  |  | X |  |  |  |  |  |
| Ishii et al., 2010 | If the carer addresses severe pain before care |  |  |  |  |  | X | X |  |
|  | If the carer addresses depression, delusions, and/or delirium before care |  |  |  |  |  |  | X |  |
| Ishii et al., 2012 | If the carer approaches the person with an appropriate communication style |  |  | X |  |  |  |  |  |
|  | If the carer knows the person's preferences and care goals |  | X |  |  |  |  |  |  |
|  | If carers learn from previous interactions, they can adapt their approach to the person |  | X |  |  |  |  |  |  |
|  | If carers address pain before care |  |  |  |  |  | X | X |  |
|  | If carers determine and address specific triggers before care |  |  |  |  |  | X | X |  |
|  | If carers use person centred showering or towel bathing |  | X |  |  |  |  |  |  |
| Jablonski et al., 2018 | If carers seek assent for mouthcare |  | X |  |  |  |  |  |  |
|  | If the carer builds rapport with the person - complementing them |  |  | X |  |  |  | X |  |
|  | If the carer uses gestures and pantomime |  |  |  | X |  |  |  |  |
|  | If the carer enters the person's reality 'judge is waiting' to a retired barrister |  |  |  |  |  |  |  |  |
|  | If the carer provides mouthcare in front of a mirror |  |  |  | X |  |  |  | X |
|  | If the carer approaches the person below or at eye level |  |  | X |  |  |  |  |  |
|  | If the carer avoids elderspeak |  |  |  |  |  | X |  |  |
|  | If the carer starts the activity and then enables the person to finish it |  |  |  |  | X |  |  |  |
|  | If the carer cues the person by using gestures, pantomimes and short, 1-step commands |  |  |  | X |  |  |  |  |
|  | If the carer uses bridging, where the older adult was asked to hold a toothbrush during mouth care |  |  |  |  |  |  |  | X |
|  | If the carer uses rescue, where a second experimental mouth-care provider replaced the first experimental mouth-care provider if care resistant behaviours were escalating |  |  |  | X |  |  |  |  |
|  | If the carer uses hand-over-hand, which involved either the older adult placing his or her hand over that of the experimental mouth-care provider, or the experimental mouth-care provider gently guiding the older adult's hands |  |  |  |  | X |  |  |  |
| Jablonski et al., 2011^a^ | If the carer approaches the resident at eye level within their visual field |  |  | X |  |  |  |  |  |
|  | If the carer smiles as they approach the person |  |  | X |  |  |  |  |  |
|  | If the carer establishes a rapport by complementing the person |  |  | X |  |  |  |  |  |
|  | If the carer uses gentle touch judiciously (test out with hand touch first before other touch) |  |  |  |  |  | X |  |  |
|  | If the carer assists with care in a quiet environment |  |  |  |  |  | X |  |  |
|  | If the carer positions the person in front of a sink |  |  |  | X |  |  |  |  |
|  | If the carer stands behind the person in front of the mirror to complete mouth care |  |  |  |  |  | X |  |  |
|  | If the carer does not use elderspeak |  |  |  |  |  | X |  |  |
|  | If the carer uses cues or polite one-step commands |  |  |  | X |  |  |  |  |
|  | If the carer uses gestures or pantomiming |  |  |  |  |  |  |  | X |
|  | If the carer promotes the person to self-care |  | X |  | X |  |  |  | X |
|  | If the carer primes by putting a toothbrush in the person's hand to promote self-care |  |  |  |  |  |  |  | X |
|  | If the carer initiates the activity and then hands it over to the person (chaining) |  |  |  |  | X |  |  |  |
|  | If the carer uses hand over hand technique guiding the person to complete the task |  |  |  |  | X |  |  |  |
|  | If the carer distracts the person (soft toy, singing, talking) |  |  |  |  |  |  |  | X |
|  | If the carer puts a care item (toothbrush) in the person’s hand while they also assist the person with another one of that item (Bridging) |  |  |  |  |  |  |  | X |
|  | If the carer gets another carer to take over when things are not going well (rescuing) |  |  |  | X |  |  |  |  |
| Jablonski et al., 2011^b^ | If the carer approaches the resident at eye level and in their visual field |  |  | X |  |  |  |  |  |
|  | If the carer provides mouthcare in a quiet environment with limited people present |  |  |  |  |  | X |  |  |
|  | If the carer establishes a rapport with the resident by engaging in affirming and simple conversation (e.g., complimenting a resident on his shirt) |  |  | X |  |  |  |  |  |
|  | If the carer uses gentle touch judiciously |  |  | X |  |  | X |  |  |
|  | If the carer smiles when interacting with the person |  |  | X |  |  |  |  |  |
|  | If the carer avoids elderspeak |  |  |  |  |  | X |  |  |
|  | If the carer distracts the person |  |  |  |  |  |  |  | X |
|  | If the carer uses bridging - having the elder hold the same item being used in mouth care by the caregiver such as a toothbrush or denture cup. This technique is similar to distraction except the items are congruent with the care being provided |  |  |  |  |  |  |  | X |
|  | If the carer uses priming - using objects from the environment to help the elder to initiate or complete mouth care. |  |  |  |  |  |  |  | X |
|  | If the carer uses chaining - the initiation of specific oral hygiene activities by the caregiver member with the expectation that the elder completes the activities. |  |  |  |  | X |  |  |  |
|  | If the carer uses hand-over-hand - the placing of the caregiver’s hands over the elder’s hands and guiding the elder’s hands |  |  |  |  | X |  |  |  |
|  | If the carer uses cueing - the use of polite, one-step commands |  |  |  | X |  |  |  |  |
|  | If the carer uses gestures and pantomiming |  |  |  |  |  |  |  | X |
|  | If the carer uses mirror-mirror - placed them before a mirror and provided mouth care by standing BEHIND the elders and reaching around to brush and floss their teeth. |  |  |  |  |  | X |  |  |
|  | If the carer uses rescuing - the replacement of one caregiver with another caregiver during any unsuccessful mouth care activity where care resistant behaviours are escalating. |  |  |  | X |  |  |  |  |
| Jablonski-Jaudon et al., 2016 | If the carer has an established bond with the person |  |  | X |  |  |  |  |  |
|  | If the carer requests help from the person |  |  |  |  | X |  |  |  |
|  | If the carer enters the person's reality - agrees and then circles back to suggest care would be beneficial |  |  |  |  |  |  |  | X |
|  | If the carer knows the person's history and enters their reality - the children are waiting |  |  | X |  |  |  |  |  |
|  | If the carer learns about the individual to establish rapport and modify one’s approach |  |  | X |  |  |  |  |  |
| James et al., 2020 | If the carer asks for advice/help - glad I’ve seen you, can you help with… |  |  |  |  | X |  |  |  |
|  | If the carer uses open, relaxed body language to show you are not a threat and want to help | X |  | X |  |  |  |  |  |
|  | If the carer uses simple, factual statements | X |  |  |  |  |  |  | X |
|  | If the carer de-escalates the person by -distract/redirect/therapeutic lies |  |  |  |  |  |  |  | X |
|  | If the carer uses an appropriate pace |  |  |  | X |  |  |  |  |
|  | If the carer leaves and makes things safe - let person calm down |  | X |  |  |  | X |  |  |
|  | If the carer avoids saying no/don’t/stop |  |  | X |  |  | X |  |  |
|  | If only one carer speaks at a time |  |  | X | X |  |  |  |  |
|  | If the carer keeps the person's hands busy - ask to hold the therapeutic doll in order to keep her hands occupied |  |  |  |  |  |  |  | X |
|  | If the carer uses a therapeutic lie consistent with life story |  |  | X |  |  | X |  | X |
|  | If preferred carers are brought in |  |  | X |  |  |  |  |  |
|  | If the carer shows the person that they can cope and want to help | X |  | X |  |  |  |  |  |
|  | If the carer talks about known topics that person likes - dog/travel/father |  |  | X |  |  |  |  |  |
|  | If the carer makes the environment more aligned to person - quiet |  |  |  |  |  | X |  |  |
|  | If the carer stays calm |  |  | X |  |  |  |  |  |
| Jensen et al., 2023 | If the carer provides a slow and mindfully present care delivery |  |  |  | X |  |  |  |  |
|  | If the carer understands the cause of the refusal |  | X |  |  |  |  | X |  |
|  | If the carer can be flexible in care delivery |  |  |  | X |  |  |  |  |
|  | If the carer takes time to help person understand |  |  |  |  |  |  |  | X |
|  | If the carer has a calm demeanour |  |  | X |  |  |  |  |  |
| Jung et al., 2024 | If the carer considers person’s speed preferences |  |  |  | X |  |  |  |  |
|  | If the carer involves the person in the activity |  |  |  |  | X |  |  |  |
|  | If the carer prioritises person's preferences |  | X |  |  |  |  |  |  |
|  | If the carer promotes self-care |  |  |  |  |  |  |  | X |
|  | If the carer engages in personal interaction rather than just task information |  |  | X |  |  |  |  |  |
|  | If the carer waits for the person to finishes their mouthful before offering more food |  |  |  | X |  |  |  |  |
| Kobayashi et al., 2021 | If the carer announces their presence |  |  | X |  |  |  |  |  |
|  | If the carer establishes a relationship through the relationship pillars (gaze, speech, and touch) |  |  | X |  |  | X |  |  |
|  | If the carer offers assistance with sitting/standing up |  |  |  | X |  |  |  |  |
|  | If the carer obtains consent for the relationship from the person receiving the care |  | X |  |  |  |  |  |  |
|  | If the carer provides care with a consistent positive emotional environment between the caregiver and the patient. |  |  | X |  |  |  |  |  |
|  | If the carer provides cognitive and mental stimulation that leaves a positive impression of the relationship and the care in the emotional memory of the person receiving it |  |  |  |  |  | X |  |  |
|  | If the carer, at the end of the interaction, says goodbyes, and a new meeting is scheduled, which prevents a feeling of abandonment | X |  |  |  |  |  |  |  |
| Konno et al., 2014 | If carers make sure person with hearing difficulties are wearing their hearing aids before playing music |  |  |  |  |  |  | X |  |
|  | If carers spend more time to give person centred care |  |  |  | X |  |  |  |  |
|  | If carers use music during mealtime and shower/bathtime (pre-recorded classical, old songs or hymns, or person's preference) |  |  | X |  |  | X |  |  |
|  | If carers use a person-centred approach to bathing - towel bathing and person-centred showering |  | X |  |  |  |  |  |  |
|  | If carers use an ability focussed approach (maximizing and maintaining a person's retained ability in physical and psychosocial functions by creating a supportive environment) |  |  |  | X |  |  |  |  |
|  | If the carer interacts regarding the natural environment |  |  | X |  |  |  |  | X |
|  | If the carer offers sweets during care |  |  | X |  |  |  |  |  |
|  | If the carer provides culturally sensitive, person-centred care on the basis of individual preferences and abilities |  |  |  | X |  |  |  |  |
| Konno et al., 2024 | If carers provide person centred bathing |  | X |  |  |  |  |  |  |
|  | If carers work within each person's reality |  |  | X |  |  | X |  | X |
|  | If the carer communicates well with the person |  |  | X |  |  |  |  |  |
| Kristensen and Peoples, 2020 | If the carer reassures the person, they will always get help if they need it | X |  | X |  |  | X |  |  |
|  | If the carer gives the person control over daily routines |  | X |  |  |  |  |  |  |
| Kutsumi et al., 2009 | If carers involve other care providers in care or ask for a substitute to avoid conflict (avoidance techniques) |  |  |  | X |  |  |  |  |
|  | If carers listen to their stories and go along with them |  |  | X |  |  |  |  |  |
|  | If carers observe what they say and do, and protect them from danger | X |  |  |  |  |  |  |  |
|  | If carers provide care in a calm voice and manner |  |  | X |  |  |  |  |  |
|  | If carers try to divert their attention and requests to other things, such as a walk or talk, to change their mood |  |  |  |  |  |  |  | X |
|  | If carers tell them that they can feel relieved and secure because you are with them (reassure) | X |  | X |  |  | X |  |  |
|  | If carers use combined techniques of listening and going along, diverting attention, and correction |  |  | X |  |  |  |  |  |
|  | If carers construct a relationship between person and themselves |  |  | X |  |  |  |  |  |
| Langley et al., 2022 | If the carer distracts the person |  |  |  |  |  |  |  | X |
|  | If the carer offers substitution |  |  |  | X |  |  |  |  |
|  | If the carer uses reward/bribery |  |  |  |  |  |  |  | X |
|  | If the carer uses imitation/mirroring |  |  |  |  |  |  |  | X |
|  | If the carer personalises care |  | X |  |  |  |  |  |  |
|  | If the carer initiates the activity and then hands it over to the person |  |  |  |  | X |  |  |  |
|  | If the carer sings, talks, or draws attention to a meaningful object |  |  |  |  |  |  |  | X |
|  | If the carer comes back later |  | X |  | X |  |  |  |  |
|  | If the carer lets the person hold the care item |  |  |  |  |  |  |  | X |
|  | If the carer uses one step commands |  |  |  | X |  |  |  |  |
|  | If the carer uses hand gestures and signalling |  |  |  |  |  |  |  | X |
|  | If the carer remains calm |  |  | X |  |  |  |  |  |
|  | If the carer creates a calm environment |  |  | X |  |  |  |  |  |
|  | If the carer softens the toothbrush with warm water and gently touches the lips |  |  |  |  |  | X |  |  |
|  | If the carer tries another carer |  |  |  | X |  |  |  |  |
|  | If the carer uses hand-over-hand |  |  |  |  | X |  |  |  |
| Levy-Storms et al., 2016 | If the carer gets the attention of the person |  |  |  |  |  |  |  | X |
|  | If the carer sits in front of the person when interacting (leans in) |  |  | X |  |  |  |  |  |
|  | If the carer makes and keeps eye contact with the person |  |  | X |  |  |  |  |  |
|  | If the carer directs and redirects the person to the activity (introduces/prompts verbal and non-verbal) |  |  |  |  |  |  |  | X |
| Luk et al., 2017 | If the carer places the person in an upright position |  |  |  | X |  |  |  |  |
|  | If the carer focusses on the person throughout the whole care activity |  |  | X |  |  |  |  |  |
|  | If the carer gives frequent reminders |  |  |  |  |  |  |  | X |
|  | If the carer removes distractions |  |  |  |  |  |  |  | X |
|  | If the carer forms a relationship with the person |  |  | X |  |  |  |  |  |
| Mahoney et al., 2016 | If the carer removes incorrect/dirty clothes (care objects) |  |  |  | X |  |  |  |  |
|  | If the carer minimizes and not emphasises errors |  |  |  |  |  | X |  |  |
|  | If the carer prompts a reward (watch a John Wayne movie) after the care activity |  |  |  |  |  |  |  | X |
|  | If the carer can make easier clothes look like the person usual clothes |  |  |  | X |  |  |  |  |
| Moniz-Cook et al., 2003 | If the carer finds out about the person's past they may notice and address unmet psychological needs such as for respect/security/occupation/distraction |  | X |  |  |  |  | X |  |
|  | If carers offer reassurance through distraction |  |  |  |  |  |  |  | X |
|  | If carers try later |  |  |  | X |  |  |  |  |
|  | If carers adapt the environment - change floor covering |  |  |  |  |  | X |  |  |
|  | If carers give gentle stroking/reassurance | X |  | X |  |  | X |  |  |
|  | If carers distract the person from the activity |  |  |  |  |  |  |  | X |
| Nagahama et al., 2022 | If carers prioritise favourite foods |  | X |  |  |  |  |  |  |
|  | If carers find a calming environment |  |  |  |  |  | X |  |  |
|  | If carers offer more palatable food |  | X |  |  |  |  |  |  |
| O'Brien et al., 2020 | If the carer requests patients act |  |  |  |  |  |  |  | X |
|  | If the carer uses entitlement in requesting how the person acts - announcements of future action and checking question 'I will' 'we're going to' is that okay? | X |  | X |  |  |  |  |  |
|  | If the carer acknowledges how easy task will be to carry out just little |  |  |  | X |  |  |  |  |
|  | Entitlement - proposal 'let's' |  |  |  |  | X |  |  |  |
|  | Ask permission - 'can I…' |  | X |  |  |  |  |  |  |
|  | Entitlement - direct instruction 'lift your leg' |  |  |  | X |  |  |  |  |
|  | Entitlement - statement of need 'I need to… is that alright?' |  | X |  |  |  |  |  |  |
|  | Request 'lift your leg' |  |  |  | X |  |  |  |  |
| O'Connor et al., 2011 | If the carer plays videotapes of loved one on an iPad asking the person to comply |  |  | X |  |  |  |  |  |
|  | If the carer plays videotapes of loved one on an iPad promoting social reassurance | X |  | X |  |  | X |  |  |
| Ostaszkiewicz et al., 2020^a^ | If the carer uses therapeutic communication |  |  | X |  |  |  |  |  |
|  | If the carer employs shared decision making |  | X |  |  |  |  |  |  |
|  | If the carer creates an authentic partnership with family members |  |  |  |  | X |  |  |  |
|  | If the carer acknowledges the personhood of the person |  | X |  |  |  | X |  |  |
|  | If the carer conducts a continence assessment |  |  |  |  |  |  | X |  |
| Ostaszkiewicz et al., 2020^b^ | If the carer ensures privacy (closes doors) |  |  |  |  |  |  | X |  |
|  | if the carer offers the person choices |  | X |  |  |  |  |  |  |
|  | If the carer ensures the person feels emotionally and physically safe during care | X |  |  |  |  |  |  |  |
|  | If the carer conveys kindness i.e. offering reassurance, showing tenderness and compassion, | X |  |  |  |  |  |  |  |
|  | If the carer is gentle, i.e. washing with care and using touch appropriately, |  |  |  |  |  | X |  |  |
|  | If the carer takes time to address the person’s needs (not rushing care) |  |  |  | X |  |  |  |  |
|  | If the carer adopts a partnership approach that includes listening to the person |  |  | X |  | X |  |  |  |
|  | If the carer picks up on verbal and nonverbal cues |  |  |  |  |  |  | X |  |
|  | If carers speak in a calm, soft tone |  |  | X |  |  |  |  |  |
|  | If carers use appropriate language (e.g.: ‘do you mind’ if as opposed to ‘I must’), |  | X |  |  |  |  |  |  |
|  | If the carer maintains eye contact |  |  | X |  |  |  |  |  |
|  | If the carer uses humour judiciously, |  |  | X |  |  |  |  |  |
|  | If the carer maintains a sense of calm and normality about the situation |  |  | X |  |  |  |  |  |
|  | If the carer uses appropriate touch |  |  |  |  |  | X |  |  |
|  | If the carer has a friendly and gentle attitude |  |  | X |  |  |  |  |  |
| Prizer and Zimmerman, 2018 | If the carer respects individual style and cultural preferences |  | X |  |  |  |  |  |  |
|  | If the carer gathers information on personal style preferences from the person with dementia and his/her family |  | X |  |  |  |  |  |  |
|  | If the carer lays out two outfits to encourage freedom of choice |  | X |  |  |  |  |  |  |
|  | If the carer uses a “dignity” or “modesty cape” to ensure the person is covered while dressing |  |  |  |  |  | X | X |  |
|  | If the carer uses clothing as a conversation starter to engage the person |  |  | X |  |  |  |  |  |
|  | If the carer engages the person by name and explain all actions before progressing |  |  | X |  |  |  |  | X |
|  | If the carer provides encouragement and positive reinforcement for as much independence as possible |  |  | X |  |  |  |  | X |
|  | If the carer simplifies routines |  |  |  | X |  |  |  |  |
|  | If the carer gives short verbal instructions (following graded levels of assistance) on dressing |  |  |  |  |  |  |  | X |
|  | If the carer uses gentle physical prompting (following graded levels of assistance) as needed |  |  |  |  |  |  |  | X |
|  | If the carer plans for sufficient time to dress |  |  |  | X |  |  |  |  |
|  | If the carer plays familiar music to encourage movement |  |  | X |  |  | X |  | X |
|  | If the carer chooses comfortable, simple (e.g., zippers and velcro instead of buttons and ties) dressing options |  |  |  | X |  | X |  |  |
|  | If the carer dresses the person in a comfortable and safe area (e.g., bathrooms may increase falls risk) | X |  |  |  |  | X |  |  |
|  | If the carer promotes privacy |  |  |  |  |  | X |  |  |
|  | If the carer provides positive reinforcement and reassurance to maintain personal dignity |  |  |  |  |  | X |  | X |
|  | If the carer encourages independence by using graded assistance as needed |  |  |  | X |  |  |  | X |
|  | If the carer helps maintain pattern of bathroom visits (e.g., on a timed schedule) |  | X |  |  |  |  |  |  |
|  | If the carer allows sufficient time; does not rush the person |  |  |  | X |  |  |  |  |
|  | If the carer allows the individual to choose mealtime and offers food choices |  | X |  |  |  |  |  |  |
|  | If the carer engages the individual in the mealtime experience/ preparation to stimulate appetite |  |  |  |  | X |  |  |  |
|  | If the carer maintains familiar dining routines |  | X |  |  |  |  |  |  |
|  | If the carer facilitates social eating with others |  |  | X |  | X |  |  |  |
|  | If the carer provides ample time to eat |  |  |  | X |  |  |  |  |
|  | If the carer provides verbal prompts/encouragement or physical cues at a slow pace |  |  |  | X |  |  |  | X |
|  | If the carer sits level, make eye contact, and speak with the person when assisting |  |  | X |  |  |  |  |  |
|  | If the carer encourages independence when possible |  |  |  |  |  |  |  | X |
|  | If the carer plays music |  |  | X |  |  | X |  | X |
|  | If the carer provides a quiet, relaxing, and homelike atmosphere; reduce distracting stimuli (e.g., phone calls or extra dishes, etc on the table) |  |  |  |  |  | X |  | X |
|  | If the carer ensures the dining area is well lit and uses coloured plates to provide contrast and make the food easier to see |  |  |  |  |  | X |  |  |
|  | If the carer helps the individual sit comfortably with good posture |  |  |  | X |  |  |  |  |
|  | If the carer provides appropriate and preferred foods |  | X |  | X |  |  |  |  |
|  | If the carer acts to promote the individual’s dignity, respect and choice |  | X | X |  |  |  | X |  |
| Rey et al., 2020 | If the physical environment is calm |  |  |  |  |  | X |  |  |
|  | If the carer addresses the person's overall discomfort (pain, cold, compromised modesty, weakness, insecurity) |  |  |  |  |  | X | X |  |
|  | If the carer stimulates self-care |  | X |  | X |  |  |  | X |
|  | If the carer does not use elderspeak |  |  |  |  |  | X |  |  |
|  | If care goes on for too long without breaks =not good |  |  |  | X |  |  |  |  |
|  | If the carers generate a relationship with the person |  |  | X |  |  |  |  |  |
|  | If carers discuss between themselves - not good |  |  | X |  |  |  |  |  |
|  | If the carer delivers care at an appropriate pace |  |  |  | X |  |  |  |  |
| Roberto et al., 2024 | If carers give the person some control over the situation |  | X |  |  |  |  |  |  |
|  | If the carer goes along with what the person said or did |  |  | X |  |  | X |  | X |
|  | If the carer redirects attention away from the situation |  |  |  |  |  |  |  | X |
|  | If the carer tells the person the truth | X |  |  |  |  |  |  |  |
|  | If the carer soothes the person verbally or physically |  |  |  |  |  | X |  |  |
| Shaw et al., 2023 | If the carer manages the person's pain before care |  |  |  |  |  | X | X |  |
| Shaw et al., 2022 | If the carer manages the person's pain |  |  |  |  |  | X | X |  |
|  | If the carer does not use elderspeak |  |  |  |  |  | X |  |  |
| Sloane et al., 2004 | If carers view behaviours as expressions of need and address the need - fear, pain, discomfort, need for control |  |  |  |  |  |  | X |  |
|  | If carers use focus on the person's comfort |  |  |  |  |  | X |  |  |
|  | If carers regulate the physical environment to maximize resident comfort |  |  |  |  |  | X |  |  |
|  | If carers cover the person with a towel to maintain warmth |  |  |  |  |  | X | X |  |
|  | If carers distract the person's attention (e.g., by providing food) |  |  |  |  |  |  |  | X |
|  | If carers provide choices |  | X |  |  |  |  |  |  |
|  | If carers use bathing products recommended by family and staff |  | X |  |  |  |  |  |  |
|  | If carers apply problem-solving approaches to identify causes and potential solutions |  |  |  |  |  |  | X |  |
|  | If carers focus on the person's preferences |  | X |  |  |  |  |  |  |
|  | If carers employ communication techniques appropriate for the person’s level of disease severity |  |  | X |  |  |  |  | X |
|  | If carers modify the shower spray |  |  |  |  |  | X |  |  |
|  | If carer use the towel bath is an in-bed method |  |  |  | X |  |  |  |  |
|  | If carers use no-rinse soap |  |  |  | X |  |  |  |  |
| Snow, 2022 | If the carer uses objects to show the person – not just say |  |  |  |  |  |  |  | X |
|  | If the carer makes a connection with the person - comments on something the person likes/in the care environment |  |  | X |  |  |  |  |  |
|  | If the carer introduces themselves |  |  | X |  |  |  |  |  |
|  | If the carer encourages participation in care |  |  |  |  |  |  |  | X |
|  | If the carer waits for the person's response before continuing |  |  |  |  |  |  |  | X |
|  | If the carer makes positive statements 'let's try.' |  |  |  |  | X |  |  |  |
|  | If the carer uses your body to show the person what they want them to do |  |  |  |  |  |  |  | X |
|  | If the carer keeps their voice calm, low, and rhythmic |  |  | X |  |  |  |  |  |
|  | If the carer greets the person by name a little way away from the person |  |  | X |  |  |  |  |  |
|  | If the carer gets down to equal to or below the persons eye level and uses eye contact | X |  | X |  |  | X |  |  |
|  | If the carer holds out their hand and when it is taken moves to the side of the person |  |  | X |  |  |  |  |  |
|  | If the carer seeks out unmet needs and addresses them |  |  |  |  |  |  | X |  |
|  | If the carer only gives small step by step instructions - a small part of the task at first “Lean forward” |  | X |  | X |  |  |  | X |
|  | If the carer holds up or points to the item they would like to use, possibly sharing in the dislike of the item or task “Could we try this?” |  |  |  |  | X |  |  | X |
|  | If the carer gives only the first piece of information, maybe offer a time frame of 1-5 minutes “It’s about time to (first task)” |  | X |  | X |  |  |  | X |
|  | If the carer offers choice - using visual cues to offer two possibilities or one choice with something else as the other option “This or that?” or “This or something else?” |  | X |  |  |  |  |  |  |
|  | If the carer compliments the person – Indicate something about them of value “You are looking really colourful today!” |  |  |  |  |  | X |  | X |
|  | If the carer uses hand under hand |  |  |  |  | X |  |  |  |
|  | If the carer asks the person to help them |  |  |  |  | X |  |  |  |
|  | If the carer uses empathetic statements: – “Sounds like you are… (provide an emotion)” – “Looks like you might… (provide a condition)” – “I’m sorry that happened, that’s not OK” – “This is hard, I hate it for you” | X |  | X |  |  | X |  | X |
| Sonde et al., 2011 | If there is more than one carer |  |  |  | X |  |  |  |  |
|  | If the carer leaves and goes back later |  | X |  |  |  | X |  |  |
|  | If the carer has time to let the person clean their own teeth |  | X |  |  |  |  |  | X |
|  | If the carer tells them what they are doing | X | X |  |  |  |  |  | X |
|  | If the carer distracts the person |  |  |  |  |  |  |  | X |
|  | If the carer takes thigs one step at a time |  |  |  | X |  |  |  |  |
|  | If the carer demonstrates the required action |  |  |  |  |  |  |  | X |
| Stanyon et al., 2019 | If the carer uses short instructions which are precise rather than vague and possible for the recipient to complete (roll over to the right) |  |  |  | X |  |  |  | X |
| Thorsen and Nielson, 2023 | If the carer talks a lot |  |  | X |  |  |  |  |  |
|  | If the carer validates the person's experience | X |  | X |  |  | X |  | X |
|  | If the carer approaches in a way to match the person's rhythm and mood that day |  |  | X | X |  |  |  |  |
|  | If the carer uses practices of presence (conversations that perhaps “go nowhere,” but are full of exchanges of words, murmurs, touches, motions, smiles and laughter) |  |  | X |  |  | X |  |  |
|  | If the carer emanates a calm present moods and tones (not a rushed or stressed tone) |  |  | X |  |  |  |  |  |
|  | If the carer interacts in a sincere way |  |  | X |  |  |  |  |  |
|  | If the carer first and foremost acknowledges social needs and rights of the person (social connection) |  |  | X |  |  |  |  |  |
|  | If the carer makes a social connection as a pre-requisite for care tasks |  |  | X |  |  |  |  |  |
|  | If the carer recognises the person with dementia as a social being in need of interaction and social care |  |  | X |  |  |  |  |  |
|  | If the carer creates a relationship based on 'being together' |  |  | X |  | X |  |  |  |
| Tosato et al., 2012 | If a carer manages the person's pain |  |  |  |  |  | X | X |  |
| Villar et al., 2022 | If carers offer food later |  | X |  | X |  |  |  |  |
|  | If carers give a food alternative (desert, different texture) |  | X |  | X |  |  |  |  |
|  | If carers swap or seek support from other carers |  |  |  | X |  |  |  |  |
|  | If carers involve relatives at mealtimes |  |  | X |  |  |  |  |  |
|  | If carers look for and mitigate triggers |  |  |  |  |  | X |  |  |
|  | If carers provide care in a quieter environment |  |  |  |  |  | X |  |  |
|  | If carers establish a link with the person |  |  | X |  |  |  |  |  |
|  | If carers find out about the person's history/preferences |  | X | X |  |  |  |  |  |
|  | If carers look for the cause |  |  |  |  |  |  | X |  |
| Volicer, 2021 | If carers improve communication, both verbal and non-verbal |  |  | X |  |  |  |  |  |
|  | If the carer provides a different type of care |  |  |  | X |  |  |  |  |
|  | If the carer makes care enjoyable |  |  | X |  |  |  |  |  |
|  | If the carer uses gentle touch / massage outside of care |  |  |  |  |  | X |  |  |
|  | If the carer makes the person feel safe | X |  |  |  |  |  |  |  |
|  | If the carer makes the person physically comfortable |  |  |  |  |  | X |  |  |
|  | If the carer allows the person to feel a sense of control |  | X |  |  |  |  |  |  |
|  | If the carer distracts the person |  |  |  |  |  |  |  | X |
|  | If the carer makes the person experience pleasure |  |  | X |  |  |  |  |  |
| Werner et al., 2002 | If the carer is the person's preferred/closest carer |  |  | X |  |  |  |  |  |
|  | If the carer calls another carer |  |  |  | X |  |  |  |  |
|  | If the carer reasons with the person |  |  |  |  |  |  |  | X |
|  | If the carer lets the care take place when the person wants |  |  |  | X |  |  |  |  |
|  | If the carer distracts the person by initiating social activities |  |  |  |  |  |  |  | X |
| Westerberg and Strandberg, 2007 | If the carer reminds the person what they are doing |  |  |  |  |  |  |  | X |
|  | If the carer enables the person to check the water temperature |  | X |  |  |  |  |  |  |
|  | If the carer is as calm as possible |  |  | X |  |  |  |  |  |
|  | If the carer encourages the person to take part in washing/dressing themselves as much as they can |  |  |  |  |  |  |  | X |
|  | If the carer and person cooperate together |  |  |  |  | X |  |  |  |
|  | If the carer dries the person quickly so they don’t get cold - haste |  |  |  | X |  |  |  |  |
|  | If the room temperature is warm |  |  |  |  |  | X |  |  |
|  | If the carer makes eye contact |  |  | X |  |  |  |  |  |
|  | If the carer distracts the person |  |  |  |  |  |  |  | X |
|  | If the carer has a firm but kind pitch of voice |  | X |  |  |  |  |  |  |
|  | If the carer uses items (soap) familiar to the person |  | X |  |  |  |  |  |  |
|  | If the carer knows the person and works to their preferences and mitigates their dislikes |  | X | X |  |  |  |  |  |
| Zimmerman et al., 2014 | If the carer approaches from the front |  |  | X |  |  |  |  |  |
|  | If the carer smiles |  |  | X |  |  |  |  |  |
|  | If the carer asks permission before starting |  | X |  |  |  |  |  |  |
|  | If the carer explains each step | X | X |  |  |  |  |  | X |
|  | If the carer is patient and repeats self if needed |  |  |  |  |  |  |  | X |
|  | If the carer gives positive feedback |  |  |  |  |  | X |  | X |
|  | If the carer establishes a routine |  |  |  | X |  |  |  |  |
|  | If the carer provides a reason for care |  |  |  |  |  |  |  | X |
|  | If the carer phases care - one thing focussed on each day |  |  |  | X |  |  |  |  |
|  | If carers use tell-show-do techniques to promote understanding |  |  |  |  |  |  |  | X |
|  | If the carer touches the body part to stimulate movement - touch the mouth, cheek, or jaw with the toothbrush to prompt to open |  |  |  |  |  |  |  | X |
|  | If the carer sings with the person |  |  | X |  |  | X |  | X |
|  | If the carer comes back at another time when the person might be more responsive |  | X |  |  |  | X |  |  |
|  | If the carer gives the person a care object to hold |  |  |  |  |  |  |  | X |
|  | If the carer reassures and rubs shoulder/arm to help the person relax |  |  |  |  |  | X |  |  |
|  | If the carer distracts the person |  |  |  |  |  |  |  | X |
|  | If the carer picks another time of day for care |  |  |  | X |  |  |  |  |
|  | If the carer checks for and addresses unmet needs - soreness, infection |  |  |  |  |  |  | X |  |
|  | If the carer provides care when the person is sitting up |  |  |  | X |  |  |  | X |
|  | If the carer gets a carer closer to the person to assist instead |  |  | X |  |  |  |  |  |
|  | If the carer invites the person to participate |  |  |  |  | X |  |  |  |
|  | If the carer addresses unmet needs - pain, fear, timing |  |  |  |  |  |  | X |  |
|  | **Number of sources contributing interventional strategies to each programme theory (out of n=71)** | **26** | **44** | **55** | **43** | **20** | **48** | **21** | **48** |

**References of included sources**

1. Amella, E.J. (2002). "Resistance at mealtimes for persons with dementia." Journal of Nutrition, Health & Aging. 6 (2), 117-122.
2. Apesoa-Varano, E.C. (2020). ""I know best:" women caring for kin with dementia." Social Science and Medicine 256(no pagination).
3. Ashida, S., T. R. Beachy, E. Killian, H. Pinho, M. Donohoe, H. Schneider and L. Marchini (2024). "An app to support oral hygiene care: Increasing attitudes, knowledge, and confidence in identifying oral health problems among caregivers of persons living with dementia." Special care in dentistry: official publication of the American Association of Hospital Dentists, the Academy of Dentistry for the Handicapped, and the American Society for Geriatric Dentistry. 15.
4. Astorga, M., D. Cruz-Sandoval and J. Favela (2023). "A Social Robot to Assist in Addressing Disruptive Eating Behaviors by People with Dementia." Robotics 12(1).
5. Backhouse, T., E. Dudzinski, A. Killett and E. Mioshi (2020). "Strategies and interventions to reduce or manage refusals in personal care in dementia: A systematic review." International journal of nursing studies 109: 103640.
6. Backhouse, T., Y. H. Jeon, A. Killett, J. Green, M. Khondoker and E. Mioshi (2024^b^). "Nurturing attentiveness: a naturalistic observation study of personal care interactions between people with advanced dementia and their caregivers." The Gerontologist. 24.
7. Backhouse, T., Y. H. Jeon, A. Killett and E. Mioshi (2022^a^). "How do family carers and care-home staff manage refusals when assisting a person with advanced dementia with their personal care?" Dementia (London, England) 21(8): 2458-2475.
8. Backhouse, T. and A. Ruston (2022). "Home-care workers' experiences of assisting people with dementia with their personal care: A qualitative interview study." Health & social care in the community 30(3): e749-e759.
9. Boersma, P., J. C. van Weert, B. van Meijel and R. M. Droes (2017). "Implementation of the Veder contact method in daily nursing home care for people with dementia: a process analysis according to the RE-AIM framework." Journal of clinical nursing 26(3-4): 436-455.
10. Bray, J., D. J. Brooker and C. Garabedian (2021). "What is the evidence for the activities of Namaste Care? A rapid assessment review." Dementia (London, England) 20(1): 247-272.
11. Buse, C. and J. Twigg (2018). "Dressing disrupted: negotiating care through the materiality of dress in the context of dementia." Sociology of health & illness 40(2): 340-352.
12. Cartwright, J., K. Roberts, E. Oliver, M. Bennett and A. Whitworth (2022). "Montessori mealtimes for dementia: A pathway to person-centred care." Dementia (London, England) 21(4): 1098-1119.
13. Chang, C. and B. L. Roberts (2008). "Cultural perspectives in feeding difficulty in Taiwanese elderly with dementia." Journal of Nursing Scholarship 40(3): 235-240.
14. Chou, W. Y., C. Waszynski, J. Kessler, Y. C. Chiang and R. J. Clarkson (2016). "Using positive images to manage resistance-to-care and combative behaviors in nursing home residents with dementia: A pilot study." Geriatric Nursing 37(3): 215-220.
15. Davidson, S. (2007). "Dementia: A systematic approach to understanding behaviour." Geriatrics and Aging 10(2): 104-107.
16. Del, C. and Z. J. Palace (2016). "'good morning mom and dad': A novel nonpharmacological approach to dementia management." Alzheimer's and Dementia 12(7 Supplement): P1068.
17. Faraday, J., C. Abley, F. Beyer, C. Exley, P. Moynihan and J. M. Patterson (2021). "How do we provide good mealtime care for people with dementia living in care homes? A systematic review of carer-resident interactions." Dementia (London, England) 20(8): 3006-3031.
18. Galindo-Garre F, Volicer L, van der Steen JT. Factors Related to Rejection of Care and Behaviors Directed towards Others: A Longitudinal Study in Nursing Home Residents with Dementia. Dement Geriatr Cogn Dis Extra. 2015 Apr 10;5(1):123-34. doi: 10.1159/000369158.
19. Gaugler, J. E., J. V. Hobday, J. C. Robbins and M. P. Barclay (2016). "Direct Care Worker Training to Respond to the Behavior of Individuals With Dementia: The CARES Dementia-Related BehaviorTM Online Program." Gerontology and Geriatric Medicine 2(no pagination).
20. Giang TA, Koh JEJ, Cheng LJ, Tang QC, Chua MJ, Liew TM, Wee SL, Yap PLK. Effects of Humanitude care on people with dementia and caregivers: A scoping review. J Clin Nurs. 2023 Jul;32(13-14):2969-2984. doi: 10.1111/jocn.16477.
21. Gilmore-Bykovskyi, A. L. (2015). "Caregiver person-centeredness and behavioral symptoms during mealtime interactions: development and feasibility of a coding scheme." Geriatric nursing (New York, N.Y.) 36(2 Supplement): S10-S15.
22. Gjellestad, A., T. Oksholm, H. Alvsvag and F. Bruvik (2022). "Autonomy conquers all: a thematic analysis of nurses' professional judgement encountering resistance to care from home-dwelling persons with dementia." BMC health services research 22(1): 749.
23. Gjellestad, A., T. Oksholm, H. Alvsvag and F. Bruvik (2023). "Trust-building interventions to home-dwelling persons with dementia who resist care." Nursing ethics 30(7-8): 975-989.
24. Graneheim, U. H., U. Isaksson, I. M. Ljung and L. Jansson (2005). "Balancing between contradictions: the meaning of interaction with people suffering from dementia and "behavioral disturbances"." International Journal of Aging & Human Development 60(2): 145-157.
25. Gutman, G., M. Karbakhsh, A. Vashisht, T. Kaur, R. Churchill and A. Moztarzadeh (2021). "Feasibility study of a digital screen-based calming device (MindfulGarden) for bathing-related agitation among LTC residents with dementia." Gerontechnology 20(2): 1-8.
26. Gutman, G., Vashisht, A, T. Kaur, R. Churchill, A. Moztarzadeh and M. Karbakhsh (2021). "Pilot Study of a Digital Screen–Based Calming Device for Managing Resistance During Morning and Evening Care of Persons With Dementia." Journal of Gerontological Nursing 47(11): 15-21.
27. Hammar, L. M., A. Emami, G. Engstrom and E. Gotell (2011). "Communicating through caregiver singing during morning care situations in dementia care." Scandinavian Journal of Caring Sciences 25(1): 160-168.
28. Hammar, L. M., A. Emami, E. Gotell and G. Engstrom (2011). "The impact of caregivers' singing on expressions of emotion and resistance during morning care situations in persons with dementia: An intervention in dementia care." Journal of Clinical Nursing 20(7-8): 969-978.
29. Hanson, L. C., N. Meeks, J. Guo, T. R. M. Alonzo, K. M. Mitchell, M. Gallagher, M. Toles, B. Harder and S. Zimmerman (2023). "Comfort First: Development and pilot testing of a web-based video training to disseminate Comfort Matters dementia care." Journal of the American Geriatrics Society 71(8): 2564-2570.
30. Henriques LVL, Dourado MARF, Melo RCCP, Tanaka LH. Implementation of the Humanitude Care Methodology: contribution to the quality of health care. Rev Lat Am Enfermagem. 2019 Jan 17;27:e3123. doi: 10.1590/1518-8345.2430-3123.
31. Ishii, S., J. E. Streim and D. Saliba (2010). "Potentially reversible resident factors associated with rejection of care behaviors." Journal of the American Geriatrics Society 58(9): 1693-1700.
32. Ishii, S., J. E. Streim and D. Saliba (2012). "A conceptual framework for rejection of care behaviors: Review of literature and analysis of role of dementia severity." Journal of the American Medical Directors Association 13(1): 11-23.
33. Jablonski, R. A., A. M. Kolanowski, A. Azuero, V. Winstead, C. Jones-Townsend and M. L. Geisinger (2018). "Randomised clinical trial: Efficacy of strategies to provide oral hygiene activities to nursing home residents with dementia who resist mouth care." Gerodontology 35(4): 365-375.
34. Jablonski, R. A., B. Therrien and A. Kolanowski (2011). "No more fighting and biting during mouth care: applying the theoretical constructs of threat perception to clinical practice." Research and theory for nursing practice 25(3): 163-175.
35. Jablonski, R. A., B. Therrien, E. K. Mahoney, A. Kolanowski, M. Gabello and A. Brock (2011). "An intervention to reduce care-resistant behavior in persons with dementia during oral hygiene: A pilot study." Special Care in Dentistry 31(3): 77-87.
36. Jablonski-Jaudon, R. A., A. M. Kolanowski, V. Winstead, C. Jones-Townsend and A. Azuero (2016). "Maturation of the MOUTh Intervention." Journal of Gerontological Nursing 42(3): 15-23.
37. James IA, Reichelt K, Moniz-Cook E, Lee K. Challenging behaviour in dementia care: a novel framework for translating knowledge to practice. The Cognitive Behaviour Therapist. 2020;13:e43. doi:10.1017/S1754470X20000434
38. Jensen, A. M., R. L. Wilson, B. D. Pedersen, L. Hounsgaard and E. B. Tingleff (2023). "Nursing care of people with dementia in an orthopaedic acute care setting: An integrative literature review." Journal of clinical nursing 32(9-10): 2298-2318.
39. Jung, D., H. Lee, E. Choi, J. Park and L. Yoo (2024). "Description of the mealtime of older adults with dementia in a long-term care facility: A video analysis." Geriatric Nursing 55: 176-182.
40. Kobayashi, M., M. Ito, Y. Iwasa, Y. Motohashi, A. Edahiro, M. Shirobe, H. Hirano, Y. Gineste and M. Honda (2021). "The effect of multimodal comprehensive care methodology training on oral health care professionals' empathy for patients with dementia." BMC medical education 21(1): 315.
41. Konno, R., H. S. Kang and K. Makimoto (2014). "A best-evidence review of intervention studies for minimizing resistance-to-care behaviours for older adults with dementia in nursing homes." Journal of advanced nursing 70(10): 2167-2180.
42. Konno R, Suzuki M, Hosomi A, Lizarondo L, Stern C. Assisted bathing of older adults with dementia: a mixed methods systematic review update. JBI Evid Synth. 2024 Apr 1;22(4):518-559. doi: 10.11124/JBIES-23-00043.
43. Kristensen, H. K. and H. Peoples (2020). "Experiences related to quality of life in people with dementia living in institutional settings - A meta-aggregation." British Journal of Occupational Therapy 83(3): 145-161.
44. Kutsumi, M., M. Ito, K. Sugiura, M. Terabe and H. Mikami (2009). "Management of behavioral and psychological symptoms of dementia in long-term care facilities in Japan." Psychogeriatrics 9(4): 186-195.
45. Langley, J., R. Wassall, A. Geddis-Regan, S. Watson, A. Verey, G. McKenna, P. Brocklehurst and G. Tsakos (2022). "Putting guidelines into practice: Using co-design to develop a complex intervention based on NG48 to enable care staff to provide daily oral care to older people living in care homes." Gerodontology. 14.
46. Levy-Storms, L., L. M. Harris and X. Chen (2016). "A Video-Based Intervention on and Evaluation of Nursing Aides' Therapeutic Communication and Residents' Agitation During Mealtime in a Dementia Care Unit." Journal of nutrition in gerontology and geriatrics 35(4): 267-281.
47. Luk, J. K., F. H. Chan, E. Hui and C. Y. Tse (2017). "The feeding paradox in advanced dementia: a local perspective." Hong Kong Medical Journal 23(3): 306-310.
48. Mahoney, D. F., D. W. Coon and C. Lozano (2016). "Latino/Hispanic Alzheimer's caregivers experiencing dementia-related dressing issues: corroboration of the Preservation of Self model and reactions to a "smart dresser" computer-based dressing aid." Digital Health 2.
49. Moniz-Cook, E., G. Stokes and S. Agar (2003). "Difficult behaviour and dementia in nursing homes: Five cases of psychosocial intervention." Clinical Psychology and Psychotherapy 10(3): 197-208.
50. Nagahama, Y., T. Ito, H. Fujishiro, H. Akutagawa, M. Okabe, H. Ohtaki, S. Tsukada and T. Fukui (2022). "Outcome of therapeutic interventions against food refusal in patients with dementia." Psychogeriatrics 22(1): 156-158.
51. O'Brien, R., S. Beeke, A. Pilnick, S. E. Goldberg and R. H. Harwood (2020). "When people living with dementia say 'no': Negotiating refusal in the acute hospital setting." Social Science and Medicine 263(no pagination).
52. O'Connor, C. M., R. Smith, M. T. Nott, C. Lorang and R. M. Mathews (2011). "Using video simulated presence to reduce resistance to care and increase participation of adults with dementia." American Journal of Alzheimer's Disease and other Dementias 26(4): 317-325.
53. Ostaszkiewicz, J., T. Dunning and V. Dickson-Swift (2020). "Translating dignity principles into practice for continence care for older people in care homes: A study protocol." Journal of advanced nursing 76(11): 3147-3154.
54. Ostaszkiewicz J, Dickson-Swift V, Hutchinson A, Wagg A. A concept analysis of dignity-protective continence care for care dependent older people in long-term care settings. BMC Geriatr. 2020 Jul 29;20(1):266. doi: 10.1186/s12877-020-01673-x.
55. Prizer, L.P., Zimmerman, S., 2018. Progressive Support for Activities of Daily Living for Persons Living With Dementia, Gerontologist, 58(1), S74-S87. https://doi.org/10.1093/geront/gnx103
56. Rey, S., P. Voyer, S. Bouchard and C. Savoie (2020). "Finding the fundamental needs behind resistance to care: Using the Fundamentals of Care Practice Process." Journal of clinical nursing 29(11-12): 1774-1787.
57. Roberto, K. A., B. R. McCann, J. Savla and R. Blieszner (2024). "Family Caregivers' Management of Behavioral Expressions of Dementia." The Gerontologist. 26.
58. Shaw, C., C. Ward, A. Williams, K. Lee and K. Herr (2023). "The Relationship Between Rejection of Care Behaviors and Pain and Delirium Severity in Hospital Dementia Care." Innovation in Aging 7(10).
59. Shaw, C. A., C. Ward, J. Gordon, K. N. Williams and K. Herr (2022). "Elderspeak communication and pain severity as modifiable factors to rejection of care in hospital dementia care." Journal of the American Geriatrics Society 70(8): 2258-2268.
60. Sloane PD, Hoeffer B, Mitchell CM, McKenzie DA, Barrick AL, Rader J, Stewart BJ, Talerico KA, Rasin JH, Zink RC, Koch GG. Effect of person-centered showering and the towel bath on bathing-associated aggression, agitation, and discomfort in nursing home residents with dementia: a randomized, controlled trial. J Am Geriatr Soc. 2004 Nov;52(11):1795-804. doi: 10.1111/j.1532-5415.2004.52501.x.
61. Snow, T. (2022). Positive approach to care. Available from: <https://teepasnow.com/> (Accessed 14.02.2025).
62. Sonde, L., A. Emami, H. Kiljunen and G. Nordenram (2011). "Care providers' perceptions of the importance of oral care and its performance within everyday caregiving for nursing home residents with dementia." Scandinavian Journal of Caring Sciences 25(1): 92-99.
63. Stanyon, M., S. Thomas, A. Gordon and A. Griffiths (2019). "Effects of care assistant communication style on communicative behaviours of residents with dementia: a systematic multiple case study." Scandinavian journal of caring sciences 33(1): 207-214.
64. The Eden Alternative, UK. (2025). Case Studies. Available from: <https://eden-alternative.co.uk/> (accessed 14.02.2025).
65. Thorsen M.K., and Nielsen M.L. (2023) Trust in interpersonal care relations between care providers and people diagnosed with dementia: An ethnographic study from a Danish welfare context. Dementia, 22(6) 1227–1240.
66. Tosato, M., A. Lukas, H. G. Van Der Roest, P. Danese, M. Antocicco, H. Finne-Soveri, T. Nikolaus, F. Landi, R. Bernabei and G. Onder (2012). "Association of pain with behavioral and psychiatric symptoms among nursing home residents with cognitive impairment: Results from the SHELTER study." Pain 153(2): 305-310.
67. Villar, F., K. Chacur, R. Serrat and M. Celdran (2022). "Resistance to Eating in People with Dementia Living in Long-term Care Facilities: Gaps between Common and Good Practices." Clinical gerontologist 45(4): 859-869.
68. Volicer, L. (2021). "Importance of Distinguishing Reactive and Proactive Aggression in Dementia Care." Journal of Geriatric Psychiatry and Neurology 34(3): 243-247.
69. Werner, P., N. Tabak, R. Alpert and R. Bergman (2002). "Interventions used by nursing staff members with psychogeriatric patients resisting care." International Journal of Nursing Studies 39(4): 461-467.
70. Westerberg, K. and S. Strandberg (2007). "Showering is more than resistance: Cognitive interview sequences in residential homes for elderly clients with dementia." Qualitative Research in Psychology 4(1-2): 15-28.
71. Zimmerman S, Sloane PD, Cohen LW, Barrick AL. Changing the culture of mouth care: mouth care without a battle. Gerontologist. 2014 Feb;54 Suppl 1:S25-34. doi: 10.1093/geront/gnt145.
